# Being a heritage speaker matters: the role of markedness in subject-verb person agreement in Italian

**DOI:** 10.3389/fpsyg.2024.1321614

**Published:** 2024-03-14

**Authors:** Grazia Di Pisa, Sergio Miguel Pereira Soares, Jason Rothman, Theodoros Marinis

**Affiliations:** ^1^Department of Linguistics, Humanities Section, University of Konstanz, Konstanz, Germany; ^2^Max Planck Institute for Psycholinguistics, Nijmegen, Netherlands; ^3^Department of Language and Culture, Faculty of Humanities, Social Sciences and Education, UiT The Arctic University of Norway, Tromsø, Norway; ^4^Faculty of Languages and Education, Nebrija University, Madrid, Spain; ^5^School of Psychology and Clinical Language Sciences, University of Reading, Reading, United Kingdom

**Keywords:** heritage bilingualism, subject-verb agreement, markedness, grammatical processing, Italian

## Abstract

This study examines online processing and offline judgments of subject-verb person agreement with a focus on how this is impacted by markedness in heritage speakers (HSs) of Italian. To this end, 54 adult HSs living in Germany and 40 homeland Italian speakers completed a self-paced reading task (SPRT) and a grammaticality judgment task (GJT). Markedness was manipulated by probing agreement with both first-person (marked) and third-person (unmarked) subjects. Agreement was manipulated by crossing first-person marked subjects with third-person unmarked verbs and vice versa. Crucially, person violations with 1st person subjects (e.g., *io* *suona la chitarra “I plays_-3rd-person_ the guitar”) yielded significantly shorter RTs in the SPRT and higher accuracy in the GJT than the opposite error type (e.g., il giornalista *esco spesso “the journalist go_-1st-person_ out often”). This effect is consistent with the claim that when the first element in the dependency is marked (first person), the parser generates stronger predictions regarding upcoming agreeing elements. These results nicely align with work from the same populations investigating the impact of morphological markedness on grammatical gender agreement, suggesting that markedness impacts agreement similarly in two distinct grammatical domains and that sensitivity to markedness is more prevalent for HSs.

## Introduction

The present study investigates the relationship between Subject-Verb (SV) person agreement and markedness in a group of adult heritage speakers (HSs) of Italian. Person information reflected in Italian verbal agreement morphology varies systematically depending on whether the subject is the speaker (*Io*, 1st-person singular), the addressee (*Tu*, 2nd-person singular), or someone else (*lo scrittore* “the writer,” 3rd-person singular) as shown in (1) where the form of the verb *scriv-ere* “to write” is inflected in the simple present for singular subjects.

(1)

**Table:** 

a.	Io	scriv**o.**
	*I*_-1st-person_	*write*_-1st-person_
b.	Tu	scriv**i.**
	*You*_-2nd-person_	*write*_-2nd-person_
c.	Lo scrittore	scriv**e.**
	*The writer*_-3rd-person_	*writes*_-3rd-person_

Different theoretical proposals have suggested a distinction between the first and second person on the one hand, and the third person, on the other (e.g., Jakobson, 1971; Silverstein, 1985; Harris, 1995; Harley and Ritter, 2002; Bejar, 2003; Carminati, 2005; McGinnis, 2005; Bianchi, 2006). This claim is based on the idea that the first and second person are specified as *participants* in the speech act, respectively the *speaker* and the *addressee*, while the third person, referring to someone who is neither the speaker nor the addressee, is considered a *nonparticipant*. This distinction relates to the construct of *markedness* claiming that morphological features are organized hierarchically and carry differential cognitive weight (e.g., Battistella, 1990; Bonet, 1995; Corbett, 2000; Cowper, 2005; Nevins et al., 2007; however, see Bejar (2003) suggesting that the cognitive load is not given by an arbitrary hierarchy separating *participants* from *nonparticipant* but it is more related to the levels of specification required for each person). Harley and Ritter (2002) suggest that the hierarchical organization of feature values reflects their relative degrees of ‘cognitive significance,’ with features higher on the hierarchy being more cognitively salient or costly than the ones below. This claim makes clear predictions for sentence processing, and in particular for the processing of pronouns, where the more cognitively heavy the features are, the stronger the prediction should be. Thus, within the person domain, the processing load for first/s person features should be greater than for third person (1st/2nd > 3rd). Furthermore, within the domain of verbal agreement, the third person is considered the unmarked feature value or ‘default’ person, which means it does not carry any special marking or distinctions as the first and second persons do as it is often used to refer to someone or something that is not directly involved in the conversation, making it the most neutral or unmarked category (e.g., Forchheimer, 1953; Benveniste, 1971; Harley and Ritter, 2002; Bianchi, 2006). First and second persons are instead considered the marked forms. Consequently, violations realized on marked items (1st/2nd person) are expected to be more disruptive or more cognitively costly to process compared to violations realized on unmarked ones (e.g., Deutsch and Bentin, 2001; Kaan, 2002; Nevins et al., 2007).

It is as yet not entirely clear how markedness distinctions impact the establishment of person dependencies online. Nonetheless, existent empirical research is consistent with the possibility that specified forms carry greater cognitive weight than their default counterparts, suggesting that first-and second-person cues are stronger than third-person ones (Carminati, 2005; Nevins, 2007, 2011; Nevins et al., 2007; Silva-Pereyra and Carreiras, 2007; Alemán Bañón and Rothman, 2019; Mancini et al., 2019; Alemán Bañón et al., 2021). Furthermore, other proposals from the psycholinguistics literature (e.g., Nevins et al., 2007; Wagers and Phillips, 2014) capitalize on the predictive value of marked features claiming that upon encountering a marked feature, the parser can generate a stronger prediction regarding upcoming agreement elements.

Moving on to literature on heritage languages (HLs), heritage speakers (HSs) show clear differences in their ability to produce and comprehend agreement in the verbal domain versus concord in the nominal domain (e.g., Fenyvesi, 2000; De Groot, 2005; Polinsky, 2006, 2018; Bolonyai, 2007; Albirini et al., 2011, 2013; Montrul et al., 2012; Benmamoun et al., 2013). Thus, morphological variability in HLs is asymmetric, affecting more the nominal than verbal domain. Studies have shown that in HSs of different languages, innovations in verbal agreement are less pronounced (Hindi: Montrul et al., 2012; Russian: Polinsky, 2006; Hungarian: Fenyvesi, 2000; De Groot, 2005; Bolonyai, 2007). Within the verbal domain, however, tense and mood in HLs may be more vulnerable (Rothman, 2007; Montrul, 2009; Giancaspro, 2017), while person and number violations cause fewer difficulties (Rodríguez and Reglero, 2015). This could possibly be related to the fact that verbal agreement is acquired early, is obligatory and evidenced frequently in available input, and is (typically) not context-dependent. Specifically, monolingual children produce target-like subject-verb agreement at a very young age, usually before age 3 y.o. in different languages (Italian: Belletti and Guasti, 2015; Guasti, 1993/1994, 2002; Pizzuto and Caselli, 1992; German: Clahsen, 1986; Clahsen and Penke, 1992) and simultaneous bilingual children demonstrate similar developmental paths (e.g., Austin, 2009).

The present study endeavors to move beyond descriptive comparison related to whether/how HSs perform compared to homeland L1-dominant counterparts (Rothman et al., 2023). After all, there is little doubt that there will be aggregate-level differences in terms of accuracy rates and reading times (RTs), likely related, at least in part, to the many co-existing factors that pertain (more) to HL acquisition/processing, including (although not limited to) linguistic proficiency, levels of literacy, age of acquisition effects, the role of lexical frequency, language dominance, frequency of use, type of input, as well as socio-motivational and individual cognitive factors (among others De Houwer, 2011; Unsworth, 2016; Kupisch and Rothman, 2018; Lloyd-Smith et al., 2020; Bice and Kroll, 2021; Keating, 2022; Pereira Soares, 2022; Sagarra and Rodriguez, 2022; Goldin et al., 2023; Jegerski and Keating, 2023; Paradis, 2023). Rather we examine the extent to which linguistic features come to bear on how HSs process SV person agreement at the group and individual level, probing for and unpacking systematicities that explain the variability we expect HSs to display. More specifically, the main question asked here is whether and how markedness modulates SV person agreement resolution during online processing.

The research reported in this study must be understood in the context of a general processing strategy, which has been observed for Italian HSs in a consistent and statistically significant way in a series of online and offline experiments reported by Di Pisa et al. (2022), Di Pisa and Marinis (2022), and Di Pisa (2023). In Di Pisa et al. (2022), we examined potential markedness effects in an online self-paced reading task and an offline grammaticality judgment task. Both tasks involved sentences with grammatical and ungrammatical noun-adjective agreement, manipulating gender markedness. Critically, only HSs showed a markedness effect, that is, they had significantly longer RTs and higher accuracy when violations were realized on feminine marked adjectives. Results were interpreted as a heightened sensitivity to functional morphology in the case of HS processing, resulting in a speed-accuracy tradeoff. Thus, these Italian HSs have qualitatively similar gender representations and processing abilities to homeland speakers but their context of acquisition and their unique pattern of HL use make them more sensitive to morphological patterns during real-time gender processing (see also Luque et al., 2023). These same effects were found in an elicited production task (Di Pisa and Marinis, 2022), where the same HS participants were asked to orally produce correct concord between the noun and the adjective, whereas they were not replicated in the homeland Italian and non-native late learner comparison groups reported on in Di Pisa (2023), indicating a greater reliance/awareness of HSs to overt morphological exponents, at least in the nominal domain.

In those studies, the generalizability beyond the nominal domain was left as an open question for future investigation; thus, *would the same effects of markedness be visible in another grammatical domain, for example, with verbal agreement that is less reliant on the acquisition of the lexicon as in true in the nominal domain, but rather relies more straightforwardly on syntactic rules?*

SV agreement is a grammatical structure that manifests similarly in Italian and German. However, its manifestation might be more complex in Italian than in German. Italian is a Romance language characterized by a rich morphological agreement system where each person number combination is uniquely identified by an inflectional suffix. In all types of verbs, person and number are phonologically marked on the verb form itself, while the presence of a subject pronoun is optional (pro-drop). Italian verbs are organized into three^1^ conjugations, −*are*, −*ere*, and-ire, according to the thematic vowel suffixed to the stem (Schwarze, 2009). Typically, a verb form contains four classes of elements in the following order: stem, thematic vowel (TV) (−*a, −e, −i*), tense/aspect/mood makers, person/number markers as exemplified in the first plural imperfect indicative of *nuotare* ‘to swim’ in (2):

(2)

*nuot + a + va + mo.*

*Lexical stem + TV + Past, Imperfective + 1st-person pl.*

‘(we) swam/were swimming’

Verbs from the first conjugation forming their third person singular on/a/, such as *parla* (“speaks”), are the most frequent in spoken Italian according to corpus data (De Mauro et al., 1993; Bellini and Schneider, 2019). Even though suffixation is the main form of SV agreement in Italian, many irregular verbs also involve variation in the verb stem, which even further distinguishes the different persons from each other [for example, the verb *andare* (to go): *vado* (1st-sg), vai (2nd-sg), *va* (3rd-sg), *andiamo* (1st-pl), *andate* (2nd-pl) and *vanno* (3rd-pl)].

In German, similarly to Italian, all verbs have distinct forms in singular and plural (Kunkel-Razum et al., 2009; Durrell, 2011). So, for the present tense, 1st-person singular has the suffix/−e/, the 2nd-person singular /−st/, whereas there is syncretism for /−t/, which can be 3rd-person singular or 2nd-person plural, and for/−en/and/−n/that can be 1st-or 3rd-person plural or the infinitive form (Clahsen, 1986; Albright and Fuß, 2012). Thus, German, just like Italian, has distinct forms for person marking, however, it also shows syncretism, which is not the case for Italian.

Although relevant research has been on the rise in recent years, relatively little is known about how HSs process their HLs in real-time, despite recent calls for the use of online methods [self-paced reading, eye-tracking, and EEG (electroencephalography)] in the field of heritage bilingualism (Bayram et al., 2021). The present study addresses this gap while combining an online self-paced reading task looking at online processing of HSs of Italian in Germany, and an offline grammaticality judgment task. The processing target was SV agreement and whether/how markedness asymmetry impacts the processing of (1a) sentences with a first-person singular subject (speaker role) compared to (1c) sentences with a third-person singular subject (default person), differing with respect to person markedness. Furthermore, we focused our attention on those variables that have been shown to play a relevant role in HL acquisition/processing of agreement, that is proficiency (Montrul, 2008; Alarcón, 2011; Bianchi, 2013; Kupisch et al., 2013; Di Pisa, 2023), patterns of language use (Lloyd-Smith et al., 2019, 2020; Pereira Soares, 2022; Di Pisa, 2023), and age of onset of bilingualism (Montrul, 2008; Bianchi, 2013; Keating, 2022; Di Pisa, 2023) to explore how these variables might affect SV agreement in HSs.

We build on two previous studies on native L1-dominant (Alemán Bañón and Rothman, 2019) and L2 learners of Spanish (Alemán Bañón et al., 2021) that have investigated the same processing target in a similar way using EEG as the main methodology. The authors predicted two possible scenarios: in line with their previous studies on gender agreement (Alemán Bañón and Rothman, 2016; Alemán Bañón et al., 2017), they predicted either the verb’s markedness to impact processing at the violating verb or, considering Nevins et al. (2007) proposal, the subject’s markedness would impact processing at the verb. Their results revealed that native speakers of Spanish were sensitive to both types of ungrammaticality (1st-person marked subject + *3rd-person unmarked verb; 3rd-person unmarked subject + *1st-person marked verb) as evidenced in robust positivities (P600) for both types of person violations. Crucially, person violations with a marked subject yielded a larger P600 than the opposite error type consistent with the possibility that, when encountering a subject with marked features, the parser generates a stronger prediction regarding the upcoming verb (e.g., Nevins et al., 2007; López Prego, 2015). L2 learners of Spanish were equally accurate in detecting both errors. However, the P600 was marginally reduced for “1st-person marked subject + *3rd-person unmarked verb” violations, suggesting that learners overused unmarked forms (third person) during online processing. These findings were more in line with McCarthy’s (2012) proposal relating to an overreliance on defaults in non-native learners. Importantly, this asymmetry mainly characterized learners with lower proficiency, suggesting that markedness awareness might be modulated by proficiency.

To the best of our knowledge, no study has investigated the effect of markedness on SV person agreement in HSs. Thus, our study aims to answer the following research questions (RQs):

RQ1. *Does markedness impact SV person agreement resolution in HSs? And if so, how do HSs compare to homeland speakers of Italian in this respect?*

Consistent with Alemán Bañón and Rothman (2019), an effect of markedness should impact SV agreement resolution. Two scenarios are possible; the first, in line with results from Alemán Bañón and Rothman (2019), predicts that both HSs and homeland speakers will be more sensitive to “1st-person marked subject + *3rd-person unmarked verb” violations as reflected in shorter RTs and higher accuracy for this type of violation. Alternatively, if McCarthy’s (2008, 2012) proposal is extendable to HSs in general or at least those with the lowest levels of proficiency, then like L2 learners they may rely more on defaults and, consequentially, shorter RTs and higher accuracy are predicted with the other type of violation (“3rd-person unmarked subject + *1st-person marked verb”). Although both are types of bilinguals who are not dominant in the targeted language of experimentation, our intention is definitively not to equate HSs and L2 learners *a priori*. After all, HSs are a subtype of native speakers (Rothman and Treffers-Daller, 2014; Wiese et al., 2022) who have acquired the HL naturalistically from birth, thus, they are not comparable to L2 learners as it relates to several crucial features, such as the age of onset of exposure, quantity and quality of input exposure, domains of language use and the like. However, recall that Di Pisa et al. (2022) found evidence in the domain of grammatical gender that the same set of HSs is highly sensitive to, if not reliant, on default forms.

RQ2. *To what extent do HL proficiency, patterns of HL use, and age of onset of bilingualism modulate how markedness will impact SV agreement resolution?*

Higher proficiency in the HL (i.e., Bianchi, 2013; Kupisch et al., 2013; Kupisch and Rothman, 2018; Di Pisa et al., 2022), as well as more HL use in different contexts (e.g., Bianchi, 2013; Di Pisa et al., 2022), should positively affect RTs and accuracy. Regarding the age of onset of bilingualism (AoO), two scenarios are possible: in line with Montrul (2008) and Keating (2022), sequential HSs could be more accurate and have faster RTs than simultaneous HSs. If Bianchi (2013) is right, then we should find no difference between the two groups of HSs, suggesting that verbal agreement is not affected by age effects.

## Materials and methods

### Participants

The participants included 54 adult HSs of Italian (*age* = 28.15; *SD* = 6.20; *range* = 18–41) living in Germany and 40 adult homeland Italian speakers (*M age* = 25.65; *SD* = 3.99; *range* = 18–39) living in Italy. The heritage group comprised 33 simultaneous bilinguals, where both languages (Italian and German) were present from birth, and 21 sequential bilinguals who had two native Italian-speaking parents and first came in contact with German in educational settings (between 3 and 6 y.o., *M age* = 1.5; *SD* = 1.97). All the HSs completed schooling in Germany and still lived there at the time of testing. In contrast, all Italian homeland speakers grew up in a monolingual environment and were living in Italy at the time of testing. All participants completed the Language and Social Background Questionnaire (LSBQ; Anderson et al., 2018). The questionnaire’s goal is to capture participants’ spoken languages usage and experience over their lifespan and across different settings and dimensions. It yields two composite scores and a variety of other language-related variables (e.g., age of onset of bilingualism, proficiency, etc.): a social score (henceforth referred to as “HL social”), related to language use in different social settings (e.g., work, emails, TV, etc.) and a home score (henceforth “HL home”) which is related to language use in the home life (e.g., language use with parents/siblings/grandparents, during infancy, etc.). For both variables, the higher the composite score, the more frequently the HL (Italian) was used in either social or home settings (see Supplementary Table S1 for further demographic information).

### Proficiency

**Figure 1:**
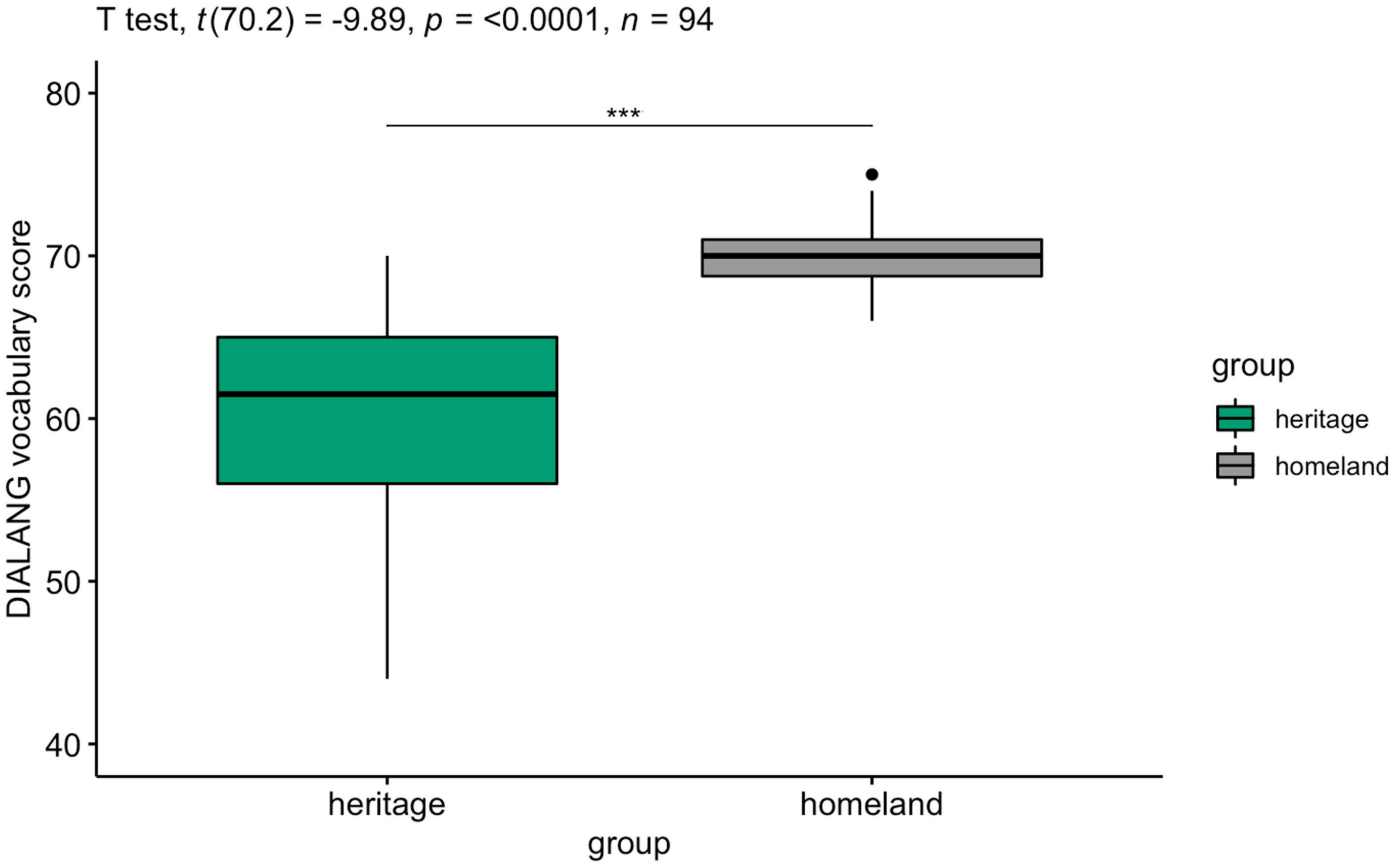
HSs and homeland speakers’ scores on the Italian vocabulary test DIALANG (raw scores). Signif. codes: 0 ‘***’, 0.001 ‘**’, 0.01 ‘*’, 0.05 ‘+’, 0.1 ‘ ’ 1.

Italian proficiency was assessed using an adapted version (Lloyd-Smith et al., 2019) of the DIALANG test battery (Alderson, 2005 for the original). The test consisted of 50 real words and 25 pseudo-words presented in the center of the screen one by one. Participants were instructed to press the F key if the word was real and J if it was not. The scoring was calculated as the sum of all correct answers both on real and non-words, leading to a total maximum possible score of 75. As shown in Figure 1, HSs exhibited lower Italian proficiency and much larger variation (*M* = 60.33; *SD* = 6.49; *range* = 44–70) than the homeland native speakers (*M* = 69.80; *SD* = 2.33; *range* = 66–75).

### Tasks

The study consisted of two main experimental tasks: a *self-paced reading task* and a *grammaticality judgment task*. To examine the contribution of person markedness to agreement we created 80 sentences of two types (see Table 1): 40 sentences had a first-person singular subject which is marked for person (condition 1) and 40 sentences had a third-person singular lexical subject which is unmarked for person (condition 3). For each of these two conditions, agreement was manipulated by pairing up first-person subjects with third-person verbs (condition 2), and third-person subjects with first-person verbs (condition 4).

**Table 1:** Sample stimuli, including the conditions examining person agreement with first-person and third-person subjects (grammatical, ungrammatical).

Condition			
1st-person marked subject		3rd-person unmarked subject	
(1)	**Marked-subject grammatical (N=20)** A | volte | io | viaggio | in | treno. Sometimes I_-1st-person_ travel_-1st-person_ by train.	(3)	**Unmarked-subject grammatical (N=20)** La | ragazza | prepara | la | torta | al | cioccolato. The girl_-3rd-person_ bakes_-3rd-person_ the chocolate cake.
(2)	**Marked-subject ungrammatical (N=20)** A | volte | io | *viaggia | in | treno. Sometimes I_-1st-person_ *travels_-3rd-person_ by train.	(4)	**Unmarked-subject ungrammatical (N=20)** La | ragazza | *preparo | la | torta | al | cioccolato. The girl_-3rd-person_ *bake_-1st-person_ the chocolate cake.

Sentences in conditions (1) and (2) follow the structure: temporal adverb *a volte* “sometimes” + subject + verb in the simple present + continuation (i.e., direct object or prepositional phrase). Sentences in condition (3) and (4) follow the structure: subject (lexical determiner phrases (DPs)) + verb in the simple present + continuation (i.e., direct object or prepositional phrase).

The same verbs (*N* = 80) were used in the conditions with first-and third-person subjects (see Supplementary Table S2 for a list of the critical verbs used in the task). Thus, at the verb (i.e., the critical word) the two markedness conditions only differed with respect to the subject. Verbs inflected for first-and third-person singular were controlled with respect to the number of characters (*M length first-person verbs*: 6.66; *SD* = 1.73; *M length third-person verbs*: 6.68; *SD* = 1.74; *t* (158) = −0.046, *p* = 0.964). In terms of frequency, third-person verbs are usually used more frequently than first-person verbs, thus the former being more frequent. Finally, the position of the critical verb was always mid-sentence, and it was similar across markedness conditions (conditions 1–2: word #4; conditions 3–4: word #3).

These materials were intermixed with 80 sentences (40 grammatical, 40 ungrammatical) from Di Pisa et al. (2022) examining noun-adjective gender agreement that did not manipulate SV agreement. These 160 sentences were counterbalanced across four experimental lists where the carrier sentences were the same. Each participant was pseudorandomly assigned to one of the four lists and the same list was used in the SPRT and the GJT, however, a given participant would not see any sentence twice within the same task.

### Self-paced reading task

HSs’ processing of SV person agreement in real-time was assessed with an online Self-Paced Reading Task (SPRT). In this task, participants’ reaction times (RTs) were measured every time they pressed the spacebar on the keyboard in order to read the sentences presented word-by-word. The 80 sentences were split into four blocks of 20 sentences, all of which contained 10 grammatical and 10 ungrammatical sentences. Short breaks were planned between the blocks. Before the actual experiment, participants were instructed on the task and four practice sentences with accuracy feedback were presented. All practice trials involved lexical materials that did not appear in the experimental stimuli. The experiment began immediately after the practice. Trials started with a fixation cross, then the first word appeared after 500 ms. A binary YES/NO comprehension question (see (3) for examples in the two grammatical conditions) was presented after 35% of the sentences on a separate display screen in order to check that participants were paying attention to the experiment. The keys F (YES) and J (NO) on their keyboard were used by the participants to respond. Participants could move to the next trial only upon response to the comprehension question (if present), and crucially no feedback was given. They were instructed to go through the words and sentences as fast as possible and were told that the task was concerned with reading comprehension. The sentences were presented in a randomized order.

(3)

- **1st-person marked subject**

A| volte| io| **viaggio**|in| treno.

R1| R2| R3| **R4**|R5| R6

| subject| **critical**|spill-over| wrap-up

“Sometimes I travel by train.”

*A volte io viaggio in macchina?*

‘Do I sometimes travel by car?’

a. *Si* “Yes.”

b. *No* “No.”

- **3rd-person unmarked subject**

La | ballerina | **corre** | ogni | giorno | al | parco.

R1 | R2 | **R3** | R4 | R5 | R6 | R7

| subject | **critical** | spill-over | wrap-up

“The dancer runs every day in the park.”

*La ballerina corre ogni giorno?*

‘Does the dancer run every day?’

a. *Si* “Yes.”

b. *No* “No.”

### Grammaticality judgment task

HSs’ accuracy in judging SV person agreement was measured with an offline Grammaticality Judgment Task (GJT). The stimuli for the GJT were exactly the same as for the SPRT, following the same experimental flow of four blocks of 20 sentences each, with 10 grammatically correct and 10 grammatically incorrect ones per block with short breaks in between. The full sentences were read by the participants on the screen and they were then asked to judge if the sentence they read was grammatically correct or not by pressing keys F (YES) or J (NO) on their keyboard. Sentences were presented in a random order.

### Procedure

The experimental session was completed entirely online by each participant using their personal computer. All tasks were created and implemented using Gorilla Experiment Builder^2^ (Anwyl-Irvine et al., 2020). The experimental session started with participants filling out the LSBQ, then they completed the DIALANG in Italian, the SPRT, and finally the GJT. The whole session lasted around 45 min. Participants were allowed to take breaks in between the tasks. Participants were compensated for their participation. Informed consent was obtained from all participants prior to the start of the experiment and all procedures were approved by the University of Konstanz research ethics committee.

### Analyses

Raw RTs were screened for extreme values and outliers (Keating and Jegerski, 2015; Marsden et al., 2018). All segments with RTs below 150 ms and above 6,000 ms were excluded (1.85% of data excluded). The remaining data were trimmed, so that raw RTs that exceeded 2.5 standard deviations below and above from the participants’ mean per position and per condition were excluded. This led to a further 3.30% of data exclusion. In total, 5.15% of the data were removed.

Sentences were segmented into 6 (1st-person subject) or 7 (3rd-person subject) regions of interest. Analyses for RTs were performed on 2 specific regions: *verb region* (critical region) and *spill-over region* (post-critical region) (see example (3) above). Since sentences in the two different conditions (1st-person subject vs. 3rd-person subject) were of different lengths, but shared the same conceptual critical regions, they were merged for analyses across verb and spillover regions. RTs from the SPRT were analyzed using mixed-effects linear models (Baayen et al., 2008), whereas accuracy data from the GJT were analyzed using mixed-effects logistic regressions of the binomial family (Jaeger, 2008). All analyses were done in R (R Core Team, 2016). The *mixed* function in the *afex* package (Singmann et al., 2022) was used to run a likelihood ratio test. Categorical variables were sum-coded, whereas numerical variables were centered around the mean. Pairwise post-hoc comparisons (Tukey contrasts) were carried out within the *emmeans* package (Lenth, 2022). Figures were created using the *ggplot2* package (Wickham, 2016).

Two types of comparative analyses were conducted. The first one focused on an HSs vs. homeland speakers comparison and sought to establish whether both groups were sensitive to verb markedness as reflected in differential RTs in the SPRT and accuracy in the GJT. The investigated dependent variables were RTs for the SPRT and a binary accuracy outcome (correct or incorrect) for the GJT. For both tasks, *Group* (heritage vs. homeland speakers), *Grammaticality* (grammatical vs. ungrammatical) and *Markedness* (marked vs. unmarked), as well as their interactions (*Group:Grammaticality, Group:Markedness, Grammaticality:Markedness, Group:Grammaticality:Markedness*) were included as fixed effects. The random effects for the SPRT included random slopes for *Subject* and random intercept for *Item* while for the GJT models, random effects included *Grammaticality* + *Markedness* slopes for *Subject* and *Grammaticality* intercept for *Item*. All models were simplified following Bates et al. (2015) suggestions until no convergence issues were outputted.

The second analysis was performed only on the HSs in order to investigate whether and how language variables (DIALANG proficiency scores, bilingualism type, LSBQ factors) predicted RTs (SPRT) and accuracy (GJT). The fixed effects included in the model were *Grammaticality* (grammatical vs. ungrammatical), *Markedness* (marked vs. unmarked), *Proficiency* (DIALANG proficiency scores centered), *Bilingualism* (simultaneous vs. sequential), HL use in the home (*HL_home*; centered) and in the society (*HL_social*; centered), as well as their interactions (*Grammaticality:Markedness, Grammaticality:Proficiency, Grammaticality:Bilingualism, Grammaticality:HL_home, Grammaticality:HL_social, Markedness:Proficiency, Markedness:Bilingualism, Markedness:HL_home, Markedness:HL_social, Grammaticality:Markedness:Proficiency, Grammaticality:Markedness:Bilingualism, Grammaticality:Markedness:HL_home, Grammaticality:Markedness:HL_social*). The random effects were built the same as for the previous models: for the SPRT, random slopes for *Subject* and random intercept for *Item*; for the GJT, random effects included *Grammaticality* + *Markedness* slopes for *Subject* and *Grammaticality* intercept for *Item*. Models were simplified until there were no convergence issues. An overview of all model specifications as well as the complete presentation of their effects for both tasks can be found in Supplementary materials 2, 3.

## Results

For the SPRT, figures and averages are reported in raw measures for ease of exposition, but the models were all fit using log-transformed RTs, in order to remove skews and to normalize model residuals (Vasishth and Nicenboim, 2016).

### Self-paced reading task

**Figure 2:**
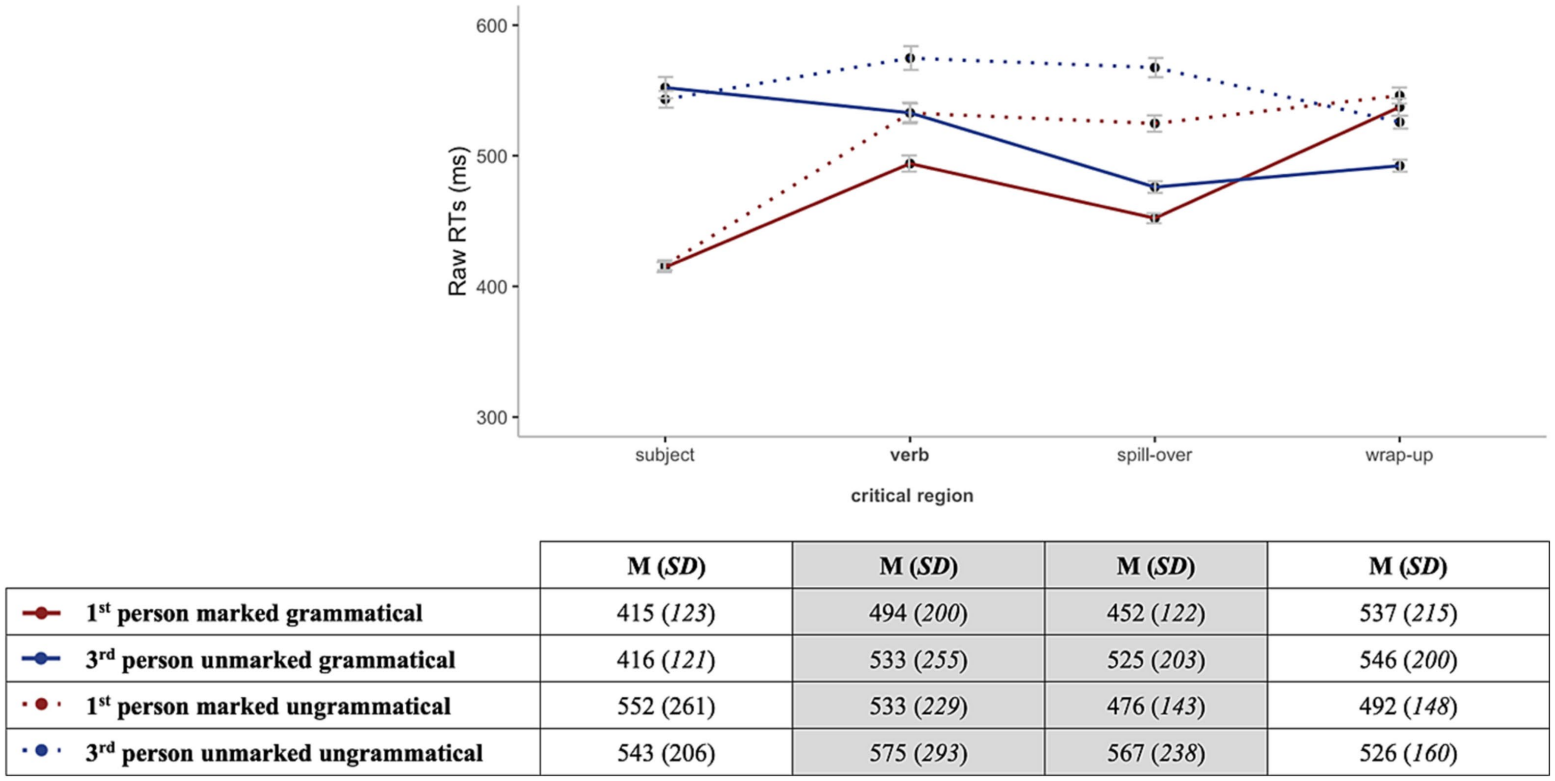
Mean RTs for the HSs by region for grammatical (solid lines) vs. ungrammatical (dotted lines) sentences for 1st-person marked (red) and 3rd-person unmarked (blue).

**Figure 3:**
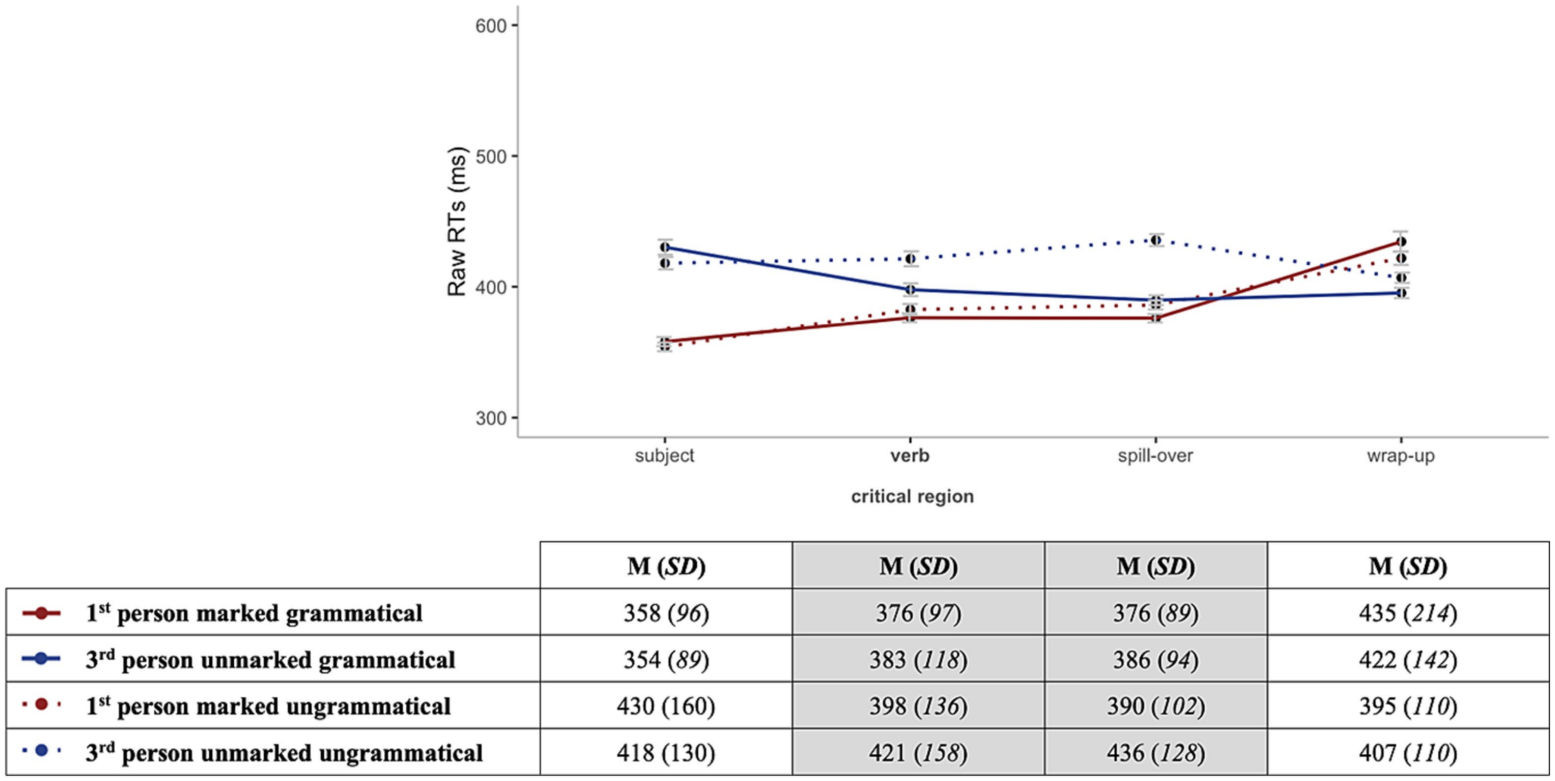
Mean RTs for the homeland speakers by region for grammatical (solid lines) vs. ungrammatical (dotted lines) sentences for 1st-person marked (red) and 3rd-person unmarked (blue).

Accuracy rates for the comprehension question responses were analyzed to make sure that participants were reading for meaning and were attentive during the task. Both groups exhibited high accuracy rates in both grammatical and ungrammatical conditions as shown in Table 2. Since all participants scored above chance (50% accuracy), no one was excluded. For the RT analysis, only trials that received correct answers were included. Figures 2, 3 illustrate overall reading patterns; as expected HSs had longer RTs than homeland speakers.

**Table 2:** Mean accuracy scores (%) and standard deviations per condition for HSs and homeland in the SPRT – comprehension accuracy.

Condition	HSs	Homeland
	M (*SD*)	M (*SD*)
1st-person marked grammatical	98 (0.13)	97 (0.17)
3rd-person unmarked grammatical	97 (0.18)	97 (0.17)
1st-person marked ungrammatical	98 (0.15)	96 (0.20)
3rd-person unmarked ungrammatical	97 (0.18)	99 (0.12)

In the **verb region** (critical), three different effects were observed. First, we found an effect of *Group* (Chisq = 18.14, *p* < 0.001), showing that HSs were reading at a slower pace as compared to the homeland speakers. Furthermore, we observed an effect of *Grammaticality* (Chisq = 26.89, *p* < 0.001) and *Markedness* (Chisq = 5.28, *p* = 0.022), indicating that the participants were faster for grammatical and 1st-person marked conditions in comparison to ungrammatical and 3rd-person unmarked conditions, respectively.

**Figure 4:**
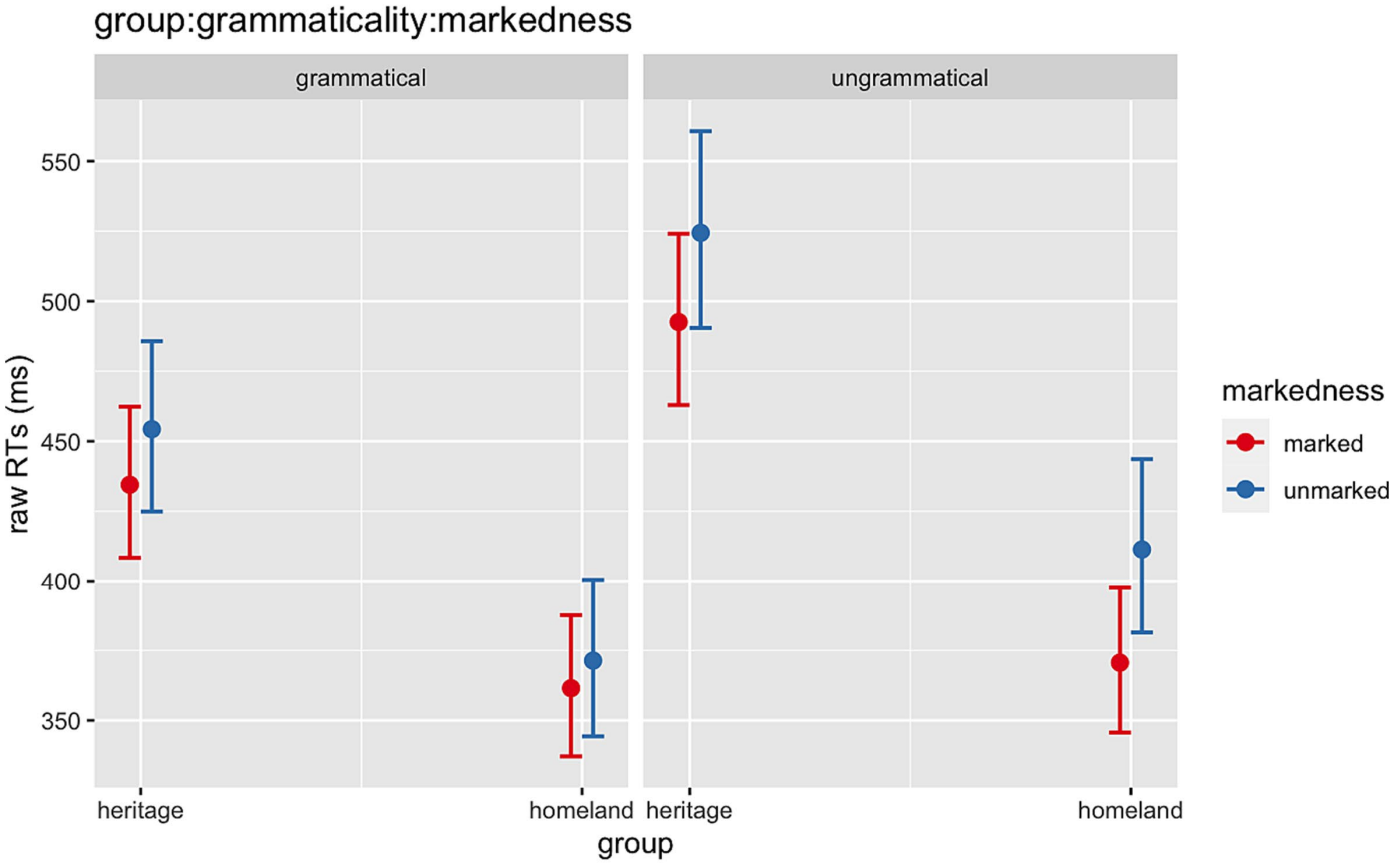
Illustration of the three-way interaction between *Group* (HSs, homeland), *Grammaticality* (grammatical, ungrammatical), *Markedness* (1st-person marked, 3rd-person unmarked).

In the **spill-over region** (post-critical), several significant effects were found. In no particular order, we observed an effect of *Group* (Chisq = 24.64, *p* < 0.001), *Grammaticality* (Chisq = 314.39, *p* < 0.001) and *Markedness* (Chisq = 6.50, *p* = 0.011), which reflect expected behaviors, i.e., HSs reading slower than homeland speakers and grammatical and 1st-person marked sentences being processed faster than ungrammatical and 3rd-person unmarked ones. Moreover, the analysis revealed the following significant interactions: *Group:Grammaticality* (Chisq = 40.73, *p* < 0.001), *Grammaticality:Markedness* (Chisq = 18.63, *p* < 0.001) and critically, a three-way interaction *Group:Grammaticality:Markedness* (Chisq = 7.16, *p* = 0.007) (Figure 4).

Post-hoc analyses on the significant interaction between *Group:Grammaticality* indicated that HSs were slower at reading both grammatical (*β* = 0.193, *SE* = 0.043, *z* = 4.448, *p* < 0.001) and ungrammatical (*β* = 0.264, *SE* = 0.043, *z* = 6.079, *p* < 0.001) sentences in comparison to the homeland speakers. Furthermore, grammatical sentences were read faster than ungrammatical ones in both groups (HSs: *β* = −0.135, *SE* = 0.007, *z* = −18.640, *p* < 0.001; homeland: *β* = −0.064, *SE* = 0.008, *z* = −7.613, *p* < 0.001). Subsequent post-hoc pairwise comparisons within the second significant interaction *Grammaticality:Markedness* showed that in the grammatical conditions there was no difference in RTs between 1st-person marked and 3rd-person unmarked (*β* = −0.036, *SE* = 0.024, *z* = −1.517, *p* = 0.130), whereas, in the ungrammatical conditions, sentences with a 1st-person marked were read faster than the ones with 3rd-person unmarked (*β* = −0.084, *SE* = 0.024, *z* = −3.543, *p* < 0.001). Finally, post-hoc tests for the three-way interaction between *Group:Grammaticality:Markedness* highlighted that for the 1st-person marked subjects, there was a difference in RTs between the grammatical vs. ungrammatical conditions for the HSs, whereas this was not the case for the homeland group (HSs: *β* = −0.125, *SE* = 0.010, *z* = −12.288, *p* < 0.001; homeland: *β* = −0.025, *SE* = 0.012, *z* = −2.118, *p* = 0.150). However, for the 3rd-person unmarked subjects, both groups showed a significant difference in RTs between grammatical vs. ungrammatical conditions. Thus, both groups showed faster RTs for the 3rd-person unmarked grammatical (HS: *β* = −0.144, *SE* = 0.010, *z* = −14.074, *p* < 0.001; homeland: *β* = −0.103, *SE* = 0.012, *z* = −8.652, *p* < 0.001).

To investigate whether proficiency and bilingual language use may affect RTs in the HSs, we fit linear mixed models to the heritage group data for verb and spillover regions.

**Figure 5:**
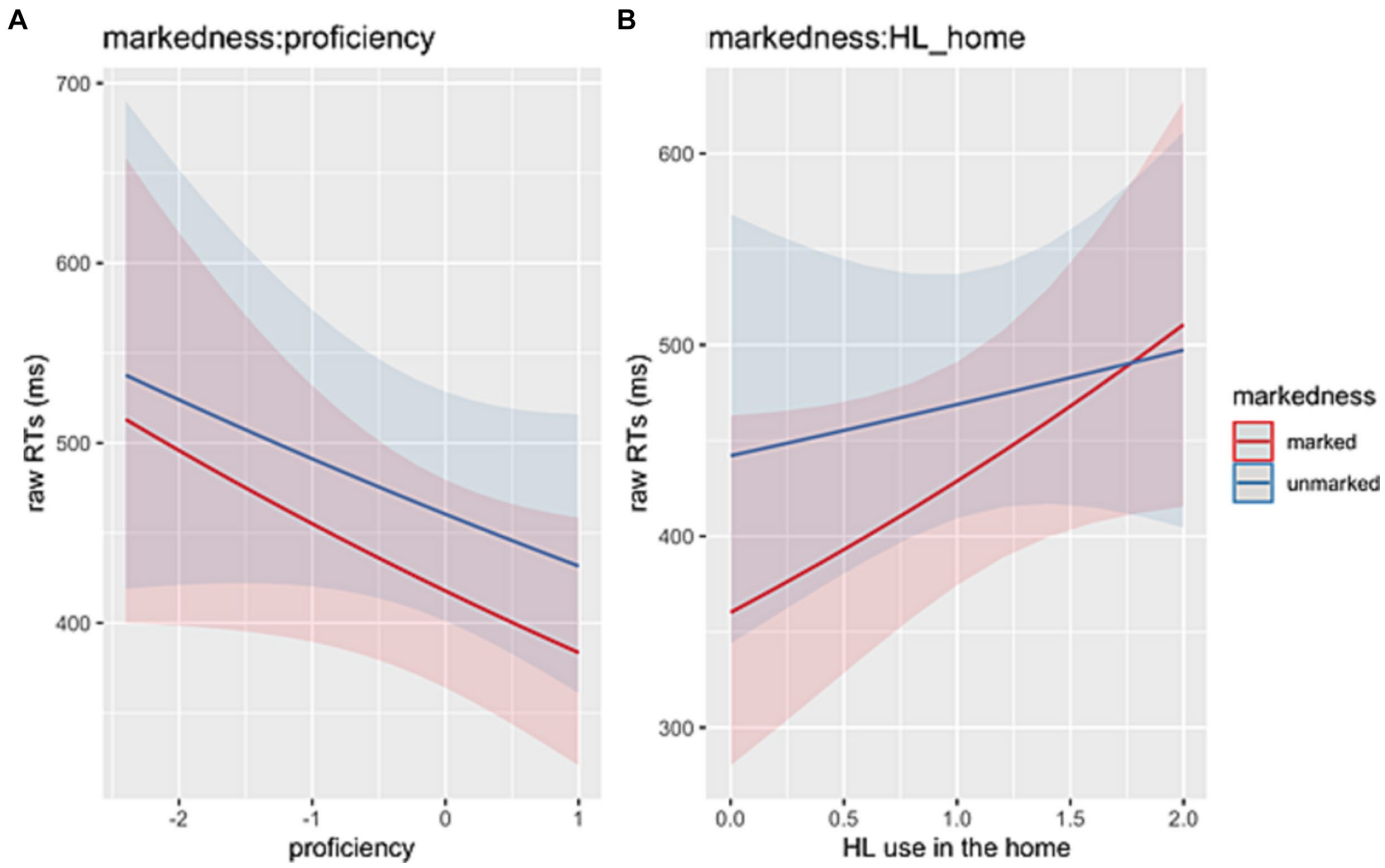
**(A)** Illustration of the two-way interactions between *Markedness* (1st-person marked, 3rd-person unmarked) and *Proficiency*. **(B)** Illustration of the two-way interactions between *Markedness* (1st-person marked, 3rd-person unmarked) and *HL use in the home*.

In the **verb region** (critical), we observed a main effect of *Markedness* (Chisq = 17.40, *p* < 0.001) reflecting faster RTs for 1st-person marked vs. 3rd-person unmarked subjects. A two-way interaction between *Markedness*:*Proficiency* (Chisq = 4.00, *p* = 0.046) indicates that HSs had shorter RTs for sentences with 1st-person marked as their proficiency increased (Figure 5A). A two-way interaction between *Markedness*:*HL_home* (Chisq = 17.89, *p* < 0.001) seems to suggest that the more the HL is used at home, the slower the HSs are in reading sentences with 1st-person marked subjects (Figure 5B).

**Figure 6:**
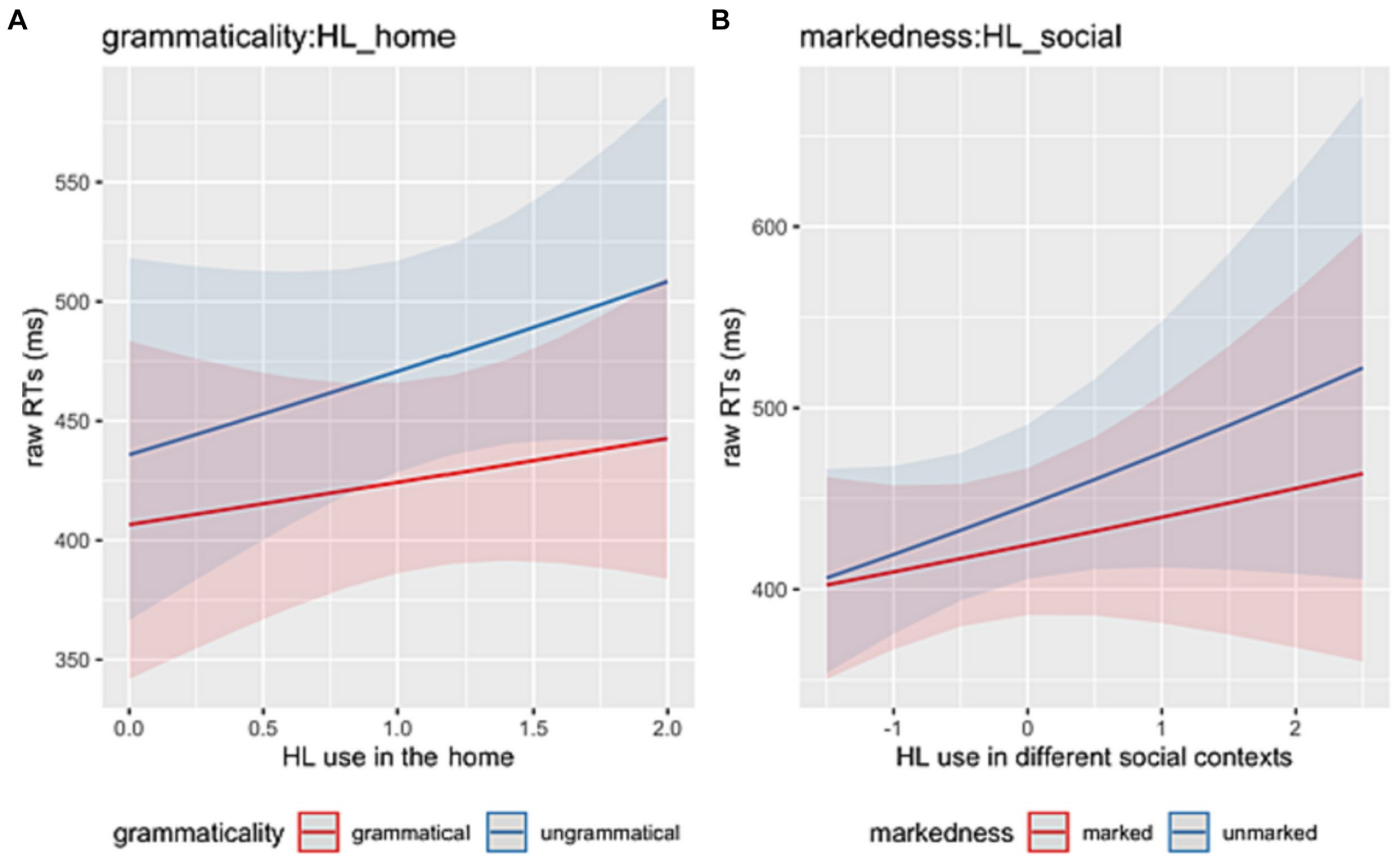
**(A)** Illustration of the two-way interactions between *Grammaticality* (grammatical, ungrammatical) and *HL use in the home*. **(B)** Illustration of the two-way interactions between *Markedness* (1st-person marked, 3rd-person unmarked) and *HL use in different social contexts*.

In the **spill-over region** (post-critical), the model revealed a main effect of *Grammaticality* (Chisq = 15.13, *p* < 0.001) indicating that HSs were sensitive to ungrammaticalities as reflected in longer RTs in the ungrammatical conditions compared to the grammatical ones. A significant two-way interaction *Grammaticality*:*HL_home* (Chisq = 8.99, *p* = 0.003) seems to indicate that the more the HL is used at home, the slower the HSs are in reading ungrammatical sentences (Figure 6A). A two-way interaction between *Markedness*:*HL_social* (Chisq = 6.63, *p* = 0.010) points to similar effects, thus the more the HL is used in different social contexts, the slower the HSs are in reading sentences with 3rd-person unmarked subjects (Figure 6B).

**Figure 7:**
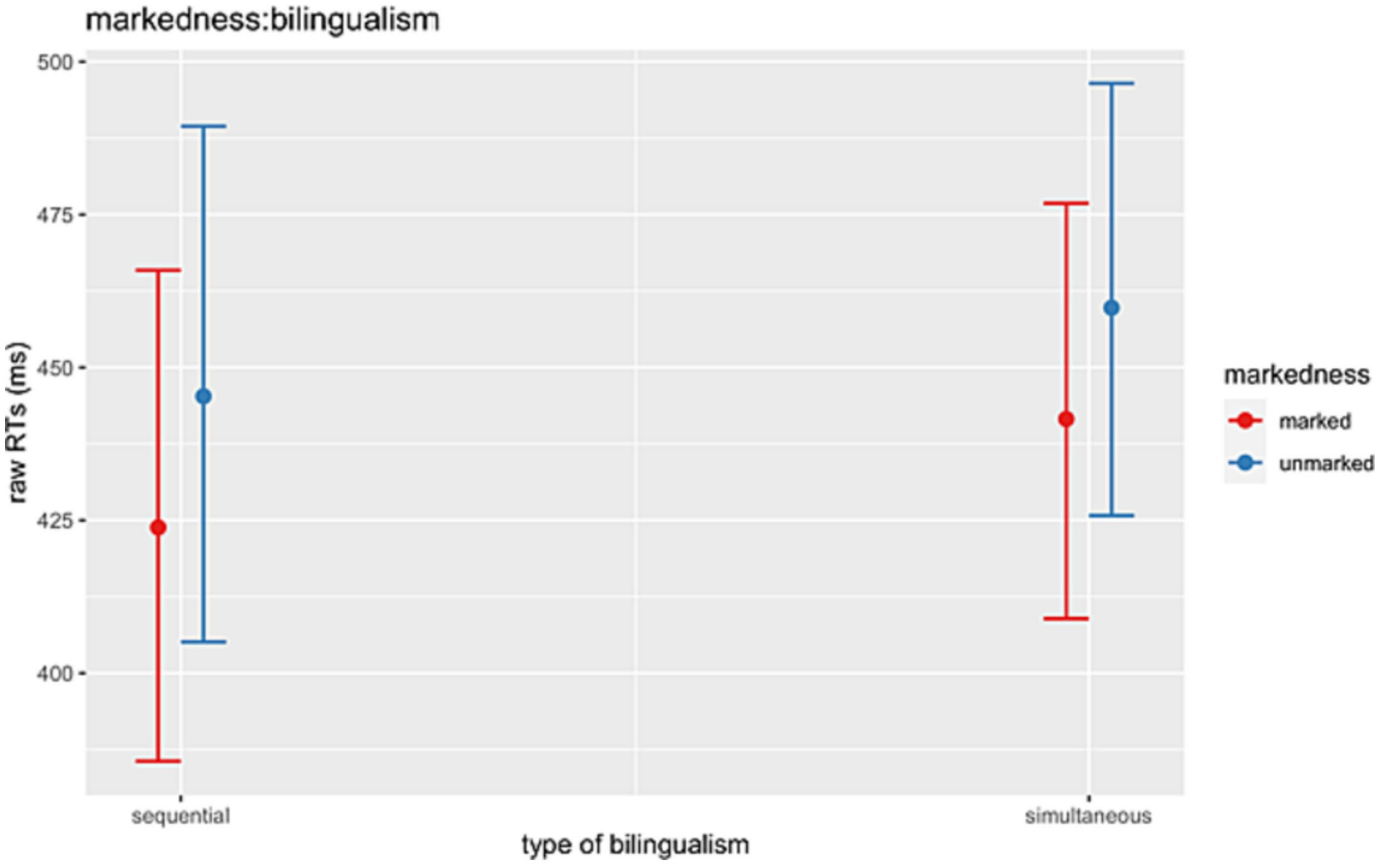
Illustration of the two-way interactions between *Markedness* (1st-person marked, 3rd-person unmarked) and *Type of bilingualism* (sequential vs. simultaneous).

Finally, a significant two-way interaction between *Markedness*:*Bilingualism* (Chisq = 4.57, *p* = 0.033) was driven by a significant effect within sequential HSs, who were faster at reading 1st-person marked than 3rd-person unmarked conditions (*β* = −0.076, *SE* = 0.026, *z* = −2.895, *p* = 0.004). This effect was not found for the simultaneous bilinguals (*β* = −0.039, *SE* = 0.025, *z* = −1.591, *p* = 0.112) (Figure 7).

In summary, the data revealed that overall HSs had longer RTs compared to homeland Italian speakers. In the critical and post-critical regions, results showed that HSs were sensitive to grammatical violations and were slower in reading ungrammatical compared to grammatical conditions. Regarding markedness, both groups were faster at reading sentences with 1st-person marked subjects versus 3rd-person unmarked ones. Finally, for the HSs, several effects relative to proficiency, HL use, and type of bilingualism suggest that the amount of language knowledge, patterns of HL use and exposure as well as the age of onset of bilingualism modulate the impact of markedness during online SV agreement.

### Grammaticality judgment task

**Figure 8:**
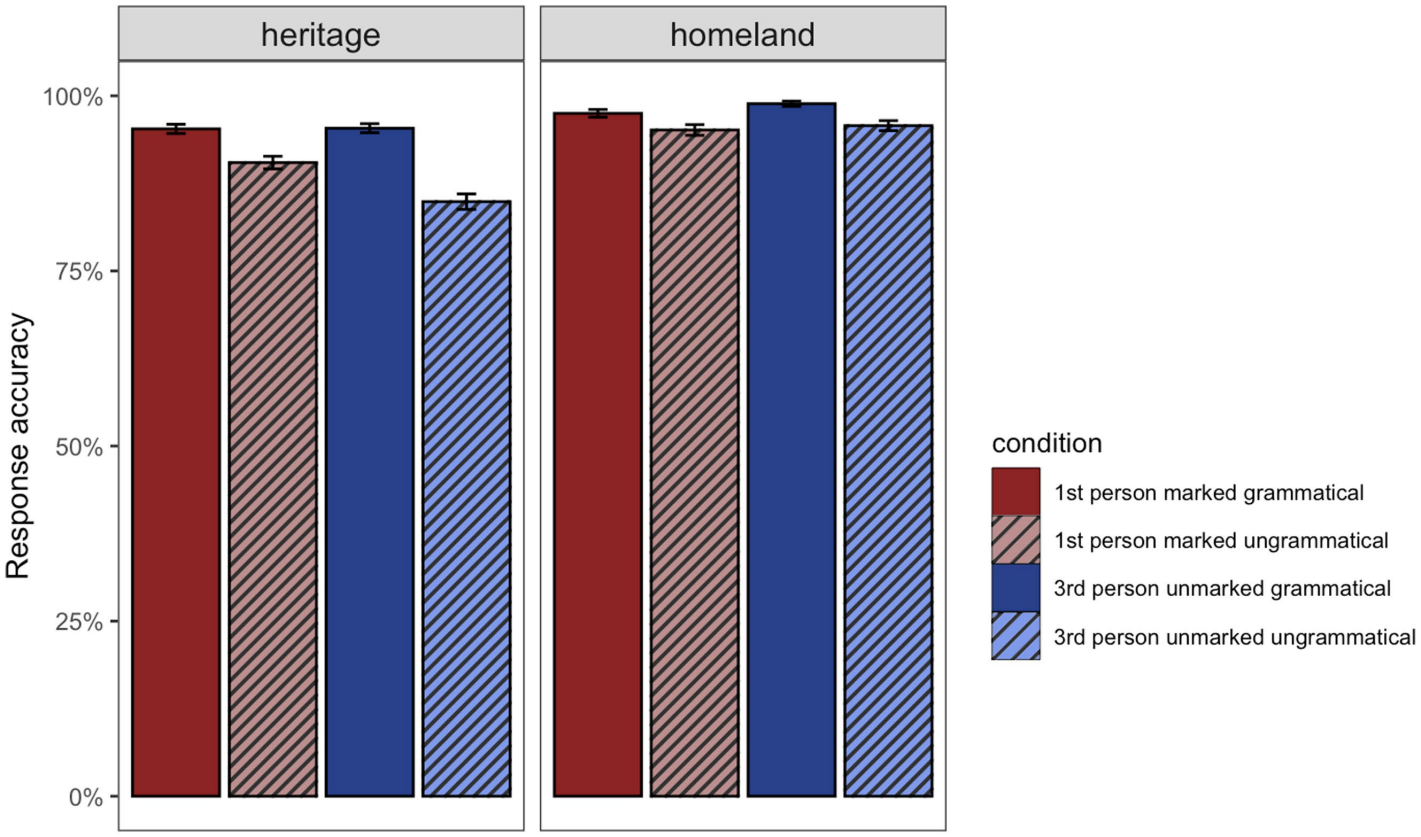
Mean response accuracy in percentage for the grammatical and ungrammatical conditions per group in the GJT. The bars represent the standard error to the mean.

The main results for the GJT are depicted in Figure 8. HSs performed with very high accuracy (95%) in both grammatical conditions, whereas in the ungrammatical conditions, accuracy in detecting violations was higher when the sentences had 1st-person marked subjects (90%) compared to sentences with 3rd-person unmarked subjects (85%). Homeland speakers’ accuracy was equal to or above 95% in all conditions.

**Figure 9:**
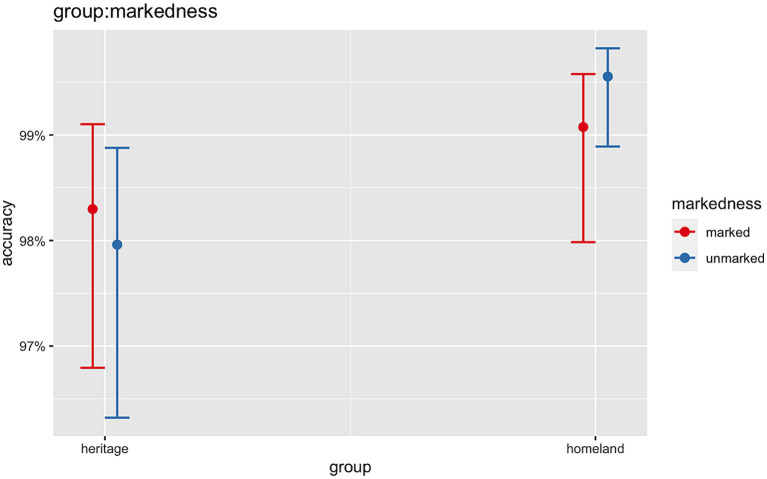
Illustration of the two-way interactions between *Group* (HSs, homeland) and *Markedness* (1st-person marked, 3rd-person unmarked).

The analyses revealed significant main effects of *Group* (Chisq = 12.98, *p* < 0.001) and *Grammaticality* (Chisq = 12.20, *p* < 0.001) showing that HSs were overall significantly less accurate as compared to homeland speakers and that in general, both groups were less accurate with ungrammatical conditions than grammatical ones. The model also revealed a significant two-way interaction between *Group:Markedness* (Chisq = 4.94, *p* = 0.026). Subsequent post-hoc pairwise comparisons showed that for the 3rd-person unmarked condition, HSs were on average significantly less accurate than their homeland peers (*β* = −1.407, *SE* = 0.341, *z* = −4.128, *p* < 0.001). This was not the case for the 1st-person marked condition (*β* = −0.557, *SE* = 0.316, *z* = −1.765, *p* = 0.08) (Figure 9).

**Figure 10:**
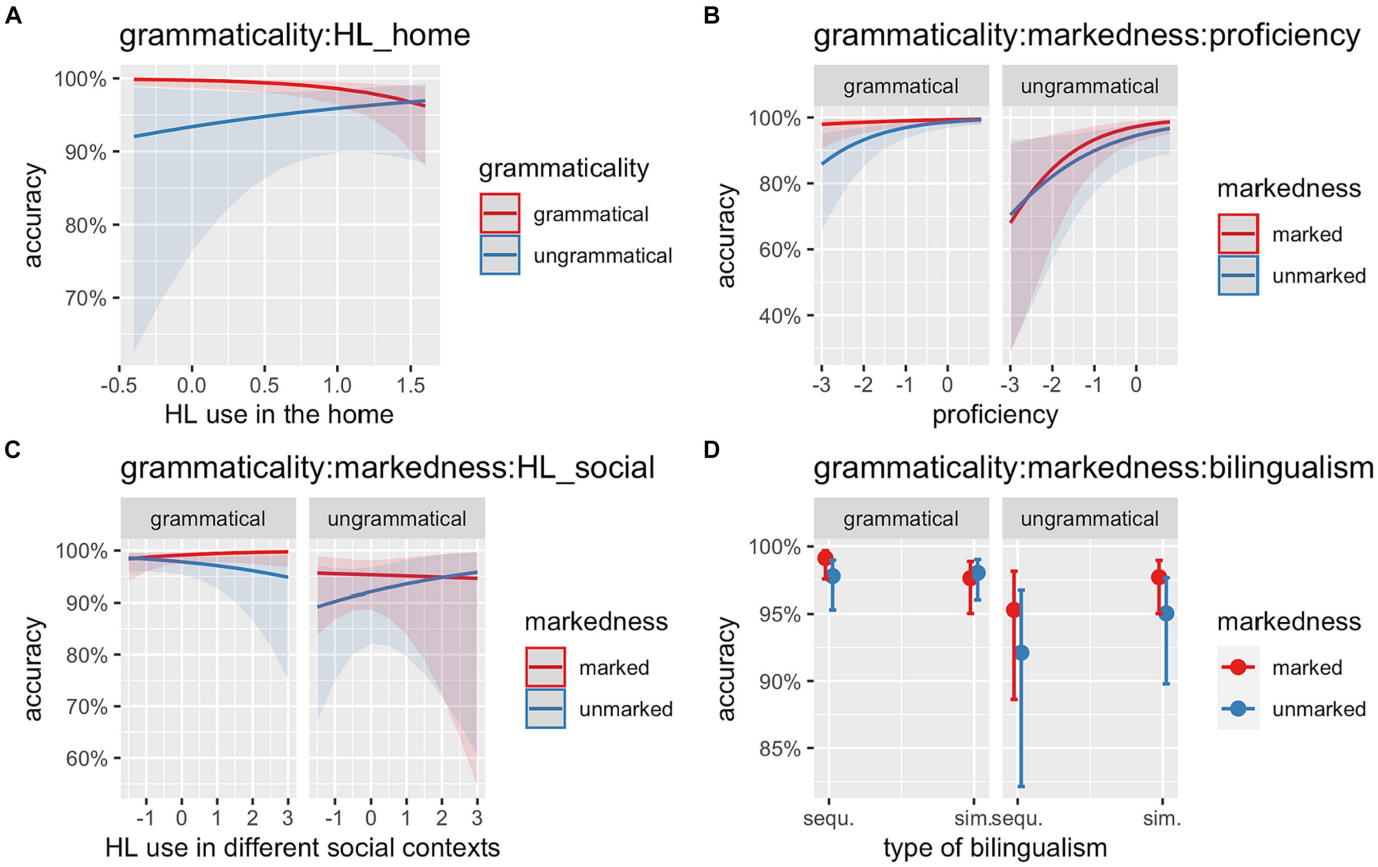
**(A)** Illustration of the two-way interaction between *Grammaticality* (grammatical, ungrammatical) and *HL use in the home*. **(B)** Illustration of the three-way interaction between *Grammaticality* (grammatical, ungrammatical), *Markedness* (1st-person marked, 3rd-person unmarked) and *Proficiency*. **(C)** Illustration of the three-way interaction between *Grammaticality* (grammatical, ungrammatical), *Markedness* (1st-person marked, 3rd-person unmarked) and *HL use in different social contexts*. **(D)** Illustration of the three-way interaction between *Grammaticality* (grammatical, ungrammatical), *Markedness* (1st-person marked, 3rd-person unmarked) and *Type of bilingualism* (sequential vs. simultaneous).

The model fitted to the HSs’ data revealed several effects. First, we observed a main effect of *Grammaticality* (Chisq = 6.85, *p* = 0.009), which indicates that HSs were more accurate in grammatical than ungrammatical conditions. Furthermore, there was a second main effect of *Proficiency* (Chisq = 15.78, *p* < 0.001), reflecting that accuracy in the task was modulated by proficiency, thus the higher the scores on the DIALANG test, the higher the accuracy in the task. A significant two-way interaction between *Grammaticality:HL_home* (Chisq = 3.98, *p* = 0.046) was found, suggesting that the more the HL is used in the home context, the higher the accuracy in the ungrammatical conditions (Figure 10A). The three-way interaction between *Grammaticality*:*Markedness:Proficiency* (Chisq = 4.95, *p* = 0.026) indicated that as proficiency in the HL increases, accuracy in both grammatical and ungrammatical conditions increases for both 1st-person marked and 3rd-person unmarked conditions. Crucially, this is more critical in the ungrammatical 1st-person marked condition (Figure 10B). The three-way interaction between *Grammaticality*:*Markedness:HL_social* (Chisq = 4.70, *p* = 0.030) seems to suggest that with more use of the HL in different social contexts, accuracy in detecting ungrammaticality with a 3rd-person unmarked subject increases (Figure 10C). Finally, the three-way interaction between *Grammaticality*:*Markedness:Bilingualism* (Chisq = 4.17, *p* = 0.041) shows that in the grammatical conditions there was no difference between the two types of bilingual, whereas in the ungrammatical conditions, sequential HSs struggled more with detecting ungrammaticality with 3rd-person unmarked subjects (Figure 10D).

To summarize, in general accuracy for HSs was lower compared to the homeland group. In terms of markedness, HSs were more accurate in detecting ungrammaticality with a 1st-person marked subject than with a 3rd-person unmarked subject. Proficiency, patterns of HL use in the home and the society as well as age of onset of bilingualism predicted accuracy in the task while modulating the impact of markedness on SV person agreement resolution.

## Discussion

The present study used an online self-paced reading task and an offline grammaticality judgment task to investigate the role of markedness in the processing of SV person agreement in a group of HSs of Italian and their homeland counterparts. Our main aim was to understand how markedness differences with respect to the speech participant status of the subject (1st-person marked vs. 3rd-person unmarked) influence online processing and offline judgments of agreement resolution at the verb. To that end, markedness was manipulated in the SV person agreement with both 1st-person (marked) and 3rd-person (unmarked) subjects (e.g., Jakobson, 1971; Harris, 1995; Harley and Ritter, 2002; Bianchi, 2006; Nevins, 2011; Alemán Bañón and Rothman, 2019; Alemán Bañón et al., 2021). Our design crossed 3rd-person singular lexical DPs subjects (*Lo scrittore* “the writer”) and 1st-person singular pronoun (*Io* “I”) with verbs inflected for the opposite person, thus two types of errors were created: “1st-person marked subject + *3rd-person unmarked verb” and “3rd-person unmarked subject + *1st-person marked verb.” Based on psycholinguistic proposals making different predictions about the role of markedness in agreement resolution, we hypothesized that it should be easier to detect a person violation realized on a 1st-person marked verb (lo scrittore *scrivo “the writer_-3rd-person_ write_-1st-person_”) because violations have been argued to be more disruptive when they are realized on marked features (e.g., Deutsch and Bentin, 2001; Kaan, 2002; Nevins et al., 2007; Alemán Bañón and Rothman, 2016). In addition, this would be in line with what we previously found for noun-adjective gender agreement in Italian with the same participants (Di Pisa et al., 2022). Alternatively, considering other proposals from the psycholinguistics literature suggesting that upon encountering a marked subject, in our case *Io* “I_-1st-person_,” the parser should generate a stronger prediction regarding the upcoming verb due to feature activation (e.g., Nevins et al., 2007; Wagers and McElree, 2011; López Prego, 2015), the violation with a 1st-person subject (Io *scrive “I_-1st-person_ writes_-3rd-person_”) should be easier to process because the subject marked status allows the parser to better resolve agreement.

Our results revealed that both groups were sensitive to agreement violations as evidenced in RT slowdowns and lower accuracy in judgment for both types of ungrammaticality. Critically, markedness affected the two groups differently in the distinct tasks. More specifically, while results from the SPRT showed that both HSs and homeland speakers were faster in reading sentences with 1st-person marked subjects versus 3rd-person unmarked ones, in the GJT, only the HSs showed an effect of markedness vis-a-vis higher accuracy for sentences with 1st-person marked subjects. We will now contextualize these results in line with data presented for the same sets of participants in Di Pisa et al. (2022) in the domain of noun-adjective gender agreement violations.

In the SPRT in Di Pisa et al. (2022), only the HSs showed a markedness effect as displayed in significantly longer RTs when the ungrammatical sentences were realized on feminine marked adjectives compared to masculine unmarked adjectives. The homeland speakers did not reveal such an effect and we attributed this to a possible ceiling effect in accuracy and/or to their very fast reading pace which could have obscured any latent effect because of the nature of self-paced reading tasks. In the present study, both groups showed a markedness effect that pointed in the same direction, thus faster RTs and higher accuracy for violation of the type “1st-person marked subject + *3rd-person unmarked.” This is in line with what Alemán Bañón and Rothman (2019) found for Spanish L1-dominant speakers and is consistent with the claim that when the parser encounters a marked subject, predictions on the upcoming verb are stronger (e.g., Carminati, 2005; Nevins et al., 2007; Wagers and McElree, 2011; López Prego, 2015). The fact that in this study we do find an effect of markedness for the homeland speakers, absent in the same cohort for gender agreement, using the same self-paced reading method could be related to the grammatical property that we are probing here. If so, these results could serve to support the view that distinct phi-features have different degrees of cognitive strength, as implied by the Feature Hierarchy (Person > Number > Gender). In other words, not all markedness relationships are the same. This claim has brought Hanson et al. (2000) and Harley and Ritter (2002) to suggest that the organization of such a geometry could account for the acquisition patterns observed in different languages, assuming that feature nodes higher in the geometry are learned sooner than those that are more deeply embedded. Thus, homeland speakers could be more sensitive to markedness asymmetries within the realm of person agreement compared to gender agreement due to the inherent nature of the feature investigated, i.e., person, which is more costly at the cognitive level compared to gender. This claim, however, would not straightforwardly explain why in the GJT we did not find similar effects. Nevertheless, it needs to be noted that accuracy in the task was equal to or higher than 95%, thus possible effects of markedness for the homeland group might have been obscured by ceiling effects in offline performance. In any case, both groups showed a pattern of results in contrast with our previous investigation on the role of markedness in the processing of noun-adjective gender agreement, involving the same participants. In Di Pisa et al. (2022), violations realized on marked adjectives yielded longer RTs and higher accuracy than violations realized on unmarked adjectives. Here, we found the reverse pattern of results suggesting that differences between the target structures probed might explain this discrepancy. It could be the case that the sentence structure we used to examine noun-adjective agreement (e.g., “*Giulio ha fotografato una **torre***_-feminine-marked_ ***antica***_-feminine-marked_ *a Londra*” “Giulio took a picture of an old tower in London”) might not have been restrictive enough to allow the parser to generate strong predictions about upcoming adjectives, since other continuations are, in principle, permissible (e.g., “*Giulio ha fotografato una **torre***_-feminine-marked_ ***che sembrava antica***_-feminine-marked_ *a Londra*” “Giulio took a picture of a tower that looked old in London”). Alternatively, for SV agreement, the presence of a subject creates a much stronger expectation that a verb phrase will follow, satisfying the minimal structure needed, SV(O), for sentence building (e.g., Chomsky, 1957, 1995). It is, therefore, possible that markedness influences agreement processing in different ways, as in being sensitive to the predictability of the dependency and the nature of the computation itself (Dillon et al., 2013).

One could claim that the parser might have extracted feature information faster and more easily from the personal pronoun *Io* “I_-1st-person_” than from the lexical DPs (*la ballerina* “the dancer_-3r-person_”), which were longer in terms of length and they might have activated other lexical information slowing down the processing of agreement. The issue of length might potentially explain the effect in the SPRT, but it would not have any weight in the GJT, where results pointed in the same direction, i.e., higher accuracy with 1st-person subjects. It is worth mentioning that the lexical DPs were not controlled in terms of lexical frequency, thus we cannot exclude the possibility that some of the DPs chosen might not have been known to the HSs. Some studies have shown that lexical frequency is a conditioning factor for processing mechanisms (e.g., Neville et al., 1992; Kutas et al., 2006), that is, less frequent words tend to slow down processing. One possibility here is that any unfamiliar DPs might have slowed down reading and influenced accuracy. However, while this could hold for HSs, it is unlikely to explain why homeland speakers also slowed down for lexical DP subjects given that it is unlikely any were unknown to them. Furthermore, considering that accuracy in the comprehension questions was very high (90% for 1st-person marked subjects and 85% for 3rd-person unmarked subjects), we are confident that the effects of markedness reported were not related to differences in lexical frequency.

Therefore, with respect to our first research question concerning whether or how markedness impacts SV agreement in HSs and homeland speakers of Italian, we see clear evidence that for the domain of verbal agreement, markedness mattered for both HSs and homeland speakers, suggesting that SV person agreement resolution in Italian is affected by the markedness status of the subject.

Our second research question sought to explore the effects of proficiency and extra-linguistic factors on the processing of SV agreement related to potential individual differences among the HSs. Similar to Di Pisa et al. (2022), a robust effect of proficiency was found to be a significant predictor of accuracy and RTs consistent with other previous studies (i.e., Bianchi, 2013; Kupisch et al., 2013). More specifically, we found that proficiency modulated the effect of markedness in terms of RTs (faster) and accuracy (higher); thus, with higher proficiency in the HL, HSs are more sensitive to the distinction between marked vs. unmarked persons. It should be noted that the DIALANG test used to assess proficiency was a measure of lexical knowledge, which provides only one dimension of an individual’s language proficiency. However, previous research has shown that lexical proficiency is a reliable measure in assessing overall language proficiency (Alderson, 2005) and positive correlations between HSs’ lexical knowledge and overall HL proficiency (i.e., Daller et al., 2003 Lloyd-Smith et al., 2019) have emphasized the importance of the lexical dimension in understanding language skills in HSs. Regarding the effect of HL use in the home and different social contexts, results from the GJT align with Di Pisa et al. (2022) and other research (Bianchi, 2013) showing that HL use plays a major role during the processing of agreement. More specifically, the present findings highlight that higher use and exposure to the HL at home and in social contexts result in smaller differences in accuracy between 1st-person marked and 3rd-person unmarked in the ungrammatical conditions. In contrast with our previous study where there was no effect of HL use in the SPRT, for verbal agreement our results showed an increase in RTs related to more use of the HL in the home and different social contexts for the ungrammatical conditions and for 3rd-person unmarked subjects. These findings might be surprising at first consideration since they seem to suggest that verbal agreement is affected negatively by more HL use/exposure. However, it could be the case that these results relate to reading effects in general or to our claims that HSs are particularly sensitive to morphological defaults (Di Pisa et al., 2022; Luque et al., 2023). That is, the more they use the language, the more they are aware of these morphological exponents. Regardless of what precisely explains directionality here, we can still observe a distinction between marked and unmarked. This sensitivity to morphology seems to be heightened for verbal agreement compared to nominal agreement, again a pattern that offers further evidence, this time from the HS performance, that distinct phi-features have different degrees of cognitive strength. Finally, in contrast to Di Pisa et al. (2022) where we found no effect of AoO of bilingualism on gender agreement, in the present study, results showed that sequential HSs were faster at reading 1st-person marked than 3rd-person unmarked conditions and they were more accurate in detecting ungrammaticality with 1st-person marked subjects. These effects were not found for the simultaneous HSs. These observations are in line with previous research on agreement (Montrul, 2008; Keating, 2022) showing that sequential HSs can be more accurate and show higher sensitivity to markedness than simultaneous HSs and suggests that AoO of bilingualism can affect grammatical processing in HSs who otherwise process agreement and have the same mental representations as their homeland peers. The fact that we found no effect of AoO for gender agreement would follow from gender being a structure acquired very early in Italian and relying more on the lexicon, whereas SV agreement relies more on syntactic rules that take longer to be acquired, and therefore, some effects of AoO can manifest themself in verbal agreement and may persist in some form in adulthood.

## Conclusion

The present study found that both HSs of Italian and homeland speakers’ use of markedness information during the processing of SV person agreement resolution was impacted by the speech participant status of the subject. Critically, person violations where the subject was the *speaker* (i.e., 1st-person marked) were processed at a faster pace and were judged more accurately than violations where the subject was not a speech participant (i.e., 3rd-person unmarked). These results were interpreted as evidence that feature activation allows the parser to generate stronger predictions regarding the upcoming verb when a marked element (i.e., 1st-person subject) is encountered (e.g., Nevins et al., 2007), otherwise, processing is costly and accuracy in detecting the violation decreases. Future studies should examine the same matter and compare the two agreement domains (nominal and verbal) in other sets of bilinguals and language combinations in order to draw stronger generalizations.

## Data availability statement

The raw data supporting the conclusions of this article will be made available by the authors, without undue reservation.

## Ethics statement

The studies involving humans were approved by the University of Konstanz Research Ethics Committee and were given a favorable ethical opinion for conduct (IRB 29/2019). The studies were conducted in accordance with the local legislation and institutional requirements. The participants provided their written informed consent to participate in this study.

## Author contributions

GP: Writing – original draft. SP: Writing – original draft. JR: Writing – review & editing. TM: Writing – review & editing.

## Footnotes

^1^Or four conjugation classes depending on how one views positioning of stress in-ere verbs, which could divide them into two unique classes.

^2^ www.gorilla.sc
