# Supplementary material for: Being a heritage speaker matters: the role of markedness in subject-verb person agreement in Italian
Source: Front Psychol. 2024 Mar 14;15:1321614. doi: 10.3389/fpsyg.2024.1321614 (PMC10972983; doi:10.3389/fpsyg.2024.1321614)
Supplement: Supplementary file 1 [file Table_1.DOCX]

**1. Supplementary Materials.**

**Table S1.** Table summarizing the indices obtained in the background questionnaire.

|  | **heritage speakers** | **homeland speakers** |
| --- | --- | --- |
|  |  |  |
|  | **M(SD)** | **M(SD)** |
|  | **Range** | **Range** |
|  |  |  |
| **Age** | 28(6.20) | 26(3.99) |
|  | 18-41 | 18-39 |
|  |  |  |
| **AoO German in years** |  |  |
| **Simultaneous (N=33)** | 0 | - |
| **Sequential (N=21)** | 1.5(1.97) | - |
|  | 3-6 | - |
|  |  |  |
| **Self-rated proficiency^1^ - range(1-40)** | 32(5.26) | 39(1.62) |
|  | 20-40 | 33-40 |
|  |  |  |
| **Speaking (max. 10)** | 8(1.40) | 10(0.66) |
|  | 4-10 | 7-10 |
|  |  |  |
| **Listening (max. 10)** | 9(1.19) | 10(0.38) |
|  | 5-10 | 8-10 |
|  |  |  |
| **Reading (max. 10)** | 8(1.71) | 10(0.33) |
|  | 4-10 | 9-10 |
|  |  |  |
| **Writing (max. 10)** | 7(1.71) | 10(0.54) |
|  | 4-10 | 8-10 |
|  |  |  |
| **Dialang score (max. score 75)** | 60(6.49) | 70(2.33) |
|  | 44-70 | 66-75 |
|  |  |  |
| **Language before 6 years old^2^** | 4(1.05) | 5(0.96) |
|  | 2-5 | 0-5 * |
|  |  |  |
| **Language after 6 years old^3^** | 4(1.05) | 5(0.99) |
|  | 1-5 | 0-5 * |
|  |  |  |
| **Language used now^4^** | 3(0.99) | 4(1.09) |
|  | 1-5 | 0-5 * |
|  |  |  |
| **Language used during school and university^5^** | 2(0.92) | 4(0.71) |
|  | 1-5 | 2-5 |
|  |  |  |
| **Italian language courses^6^**  **(N=39 participants reported to have done Italian courses)** |  |  |
| **Number of courses** | 3(1.85) | - |
|  | 1-5 | - |
| **Length of the longest course** | 4(1.41) | - |
|  | 1-5 | - |
| **Frequency of the longest course** | 2(1.33) | - |
|  | 1-5 | - |
| **Tot. number of courses in years** | 3(1.46) | - |
|  | 1-5 | - |
|  |  |  |
| **Current conversation habit^7^** | 4(1.13) | 4(0.97) |
|  | 3-6 | 3-6 |
|  |  |  |
| **Conversation habit in the past^8^** | 3(1.18) | 4(0.76) |
|  | 1-5 | 3-6 |
|  |  |  |
| **HL/Italian use in daily life^9^** | 3(0.56) | 4(0.78) |
|  | 2-4 | 1-5 * |
|  |  |  |
| **Quality of HL^10^** | 2(0.62) | 4(0.62) |
|  | 1-4 | 3-5 |
|  |  |  |
| **HL use in the home (LSBQ)^11^** | 10.85(3.93) | -3.11(5.13) ** |
|  | 3.17-18.94 | -9.86-9.92 |
|  |  |  |
| **HL use in the society (LSBQ)^12^** | 13.97(10.42) | 11.62(11.88) ** |
|  | -1.69-47.97 | -6.94-44.69 |
|  |  |  |

* Some Homeland Italian speakers considered their dialect as an “other language”.

** The Homeland Italian speakers were not monolingual (although they all grew up monolingually in Italy), thus in the LSBQ they reported the use of a L2 compared to Italian (N=30 participants reported English as L2; N=4 participants reported Spanish as L2; N=3 participants reported French as L2; N=2 participants reported German as L2; N=1 participants reported Russian as L2).

^1^Self-rated proficiency: 10 points for each language skill (speaking; listening; reading; writing).

^2^Language used at home before 6 years old: other language=0; only German=1; mainly German=2; half German/half Italian=3; mainly Italian=4; only Italian=5.

^3^Language used at home after 6 years old: other language=0; only German=1; mainly German=2; half German/half Italian=3; mainly Italian=4; only Italian=5.

^4^Language used at home now: other language=0; only German=1; mainly German=2; half German/half Italian=3; mainly Italian=4; only Italian=5.

^5^Language used during school and university: Language used in ‘elementary school’, middle school’, high school’, ‘Bachelor’, ‘Master’, PhD’; for each level only German=1; mainly German=2; half German/half Italian=3; mainly Italian=4; only Italian=5.

^6^ Italian language courses:

Number of courses: 1= 1 course; 2= 2 courses; 3= 3 courses; 4= 4 courses; 5= 5 or more courses.

Length of the longest course: 1= 1 month or less; 2= more than 1 month; 3= more than 6 months; 4= more than 1 year; 5= more than 3 years.

Frequency of the longest course: 1 = 1 course per week; 2 = 2 courses per week; 3 = 3 courses per week; 4 = 4 courses per week; 5 = 5 courses per week.

Tot. number of courses in years: 1= one year or less; 2= 2 or 3 years; 3= 4-7 years; 4= 8-11 years; 5= 12 or more years.

^7^Currrent conversation habit: No. of people Italian is spoken with – currently: 0 person=1; 1 person=2; 2-5 people=3; 6-10 people=4; 11-20 people=5; 20+ people=6.

^8^Conversation habit in the past: No. of people Italian was spoken with – in the past: 1 person= 1; 2-5 people=2; 6-10 people=3; 11-20 people=4; 20+ people=5.

^9^HL use in daily life: Language used with mother, father, siblings, grandparents, partner, friends, flat-mates, colleagues; only German=1; mainly German=2; half German/half Italian=3; mainly Italian=4; only Italian=5.

^10^Quality of HL: Language used at work, at the university, for social activities, for writing emails and texts, for watching movies and listening to music, for internet, for expressing emotions, for calculating, for reading; only German=1; mainly German=2; half German/half Italian=3; mainly Italian=4; only Italian=5.

From the LSBQ we used two factor scores:

^11^HL use in the home (possible range: –13.9 to 24.163, the higher the score, the more the HL is used in home settings)

^12^HL use in society (possible range: –7.5 to 80.304, the higher the score, the more frequently the HL is used in social settings)

For both factors, a higher score indicates more use of the HL (Italian)/L2 for homeland speakers, while a lower score indicates more use of the ML (German)/ Italian for homeland speakers.

**Table S2. List of critical verbs used in the tasks.**

|  | **Infinite form** | **Translation** |
| --- | --- | --- |
| 1 | accarezzare | *to pet* |
| 2 | accompagnare | *to bring* |
| 3 | aiutare | *to help* |
| 4 | applaudire | *to clap* |
| 5 | arrivare | *to arrive* |
| 6 | arrossire | *to blush* |
| 7 | ascoltare | *to listen* |
| 8 | atterrare | *to land* |
| 9 | ballare | *to dance* |
| 10 | bere | *to drink* |
| 11 | campeggiare | *to camp* |
| 12 | cantare | *to sing* |
| 13 | cenare | *to have dinner* |
| 14 | chiamare | *to call* |
| 15 | chiedere | *to ask* |
| 16 | comprare | *to buy* |
| 17 | controllare | *to check* |
| 18 | correggere | *to correct* |
| 19 | correre | *to run* |
| 20 | cucinare | *to cook* |
| 21 | descrivere | *to describe* |
| 22 | dimenticare | *to forget* |
| 23 | dipingere | *to paint* |
| 24 | dire | *to say* |
| 25 | disegnare | *to draw* |
| 26 | dormire | *to sleep* |
| 27 | finire | *to finish* |
| 28 | fumare | *to smoke* |
| 29 | giocare | *to play* |
| 30 | guardare | *to watch* |
| 31 | insegnare | *to teach* |
| 32 | interrogare | *to question* |
| 33 | intervistare | *to interview* |
| 34 | intimidire | *to intimidate* |
| 35 | invitare | *to invite* |
| 36 | lavorare | *to work* |
| 37 | leggere | *to read* |
| 38 | mangiare | *to eat* |
| 39 | meditare | *to meditate* |
| 40 | mentire | *to lie* |
| 41 | nuotare | *to swim* |
| 42 | obbedire | *to obey* |
| 43 | offrire | *to offer* |
| 44 | parlare | *to speak* |
| 45 | partire | *to start* |
| 46 | passeggiare | *to have a walk* |
| 47 | pattinare | *to skate* |
| 48 | perdere | *to lose* |
| 49 | piangere | *to cry* |
| 50 | piantare | *to plant* |
| 51 | pranzare | *to have lunch* |
| 52 | pregare | *to pray* |
| 53 | premiare | *to reward* |
| 54 | prendere | *to take* |
| 55 | preparare | *to prepare* |
| 56 | presentare | *to present* |
| 57 | pubblicare | *to publish* |
| 58 | pulire | *to clean* |
| 59 | punire | *to punish* |
| 60 | raccogliere | *to gather* |
| 61 | recitare | *to play a part* |
| 62 | ricevere | *to receive* |
| 63 | riflettere | *to reflect* |
| 64 | rimproverare | *to scold* |
| 65 | ripetere | *to repeat* |
| 66 | rispondere | *to answer* |
| 67 | scrivere | *to write* |
| 68 | sentire | *to feel* |
| 69 | servire | *to serve* |
| 70 | sorridere | *to smile* |
| 71 | spedire | *to send* |
| 72 | spolverare | *to dust off* |
| 73 | studiare | *to study* |
| 74 | suonare | *to play an instrument* |
| 75 | tagliare | *to cut* |
| 76 | tradire | *to betray* |
| 77 | uscire | *to go out* |
| 78 | vendere | *to sell* |
| 79 | vestire | *to dress* |
| 80 | viaggiare | *to travel* |

**2. Model specifications for the statistical analyses (SPRT)**

**Between groups**

**Critical region (Verb)**

Model:

*(log)rt ~ group + grammaticality + markedness + group:grammaticality + group:markedness + grammaticality:markedness + group:grammaticality:markedness + (1 | subject) + (1 | item)*

**Table 2.1** Model output.

| **Model output for RTs in the SPRT – Critical region verb** | | | | |  |
| --- | --- | --- | --- | --- | --- |
| *Effect* | *df* | *Chisq* | *p* |  |  |
| group | 1 | 18.14 | **<.001** | ******* |  |
| grammaticality | 1 | 26.89 | **<.001** | ******* |  |
| markedness | 1 | 5.28 | **.022** | * |  |
| group:grammaticality | 1 | 2.15 | .143 |  |  |
| group:markedness | 1 | .41 | .521 |  |  |
| grammaticality:markedness | 1 | .98 | .322 |  |  |
| group:grammaticality:markedness | 1 | 2.35 | .125 |  |  |
| Signif. codes: 0 ‘***’, 0.001 ‘**’, 0.01 ‘*’, 0.05 ‘+’, 0.1 ‘ ’ 1 |  |  |  |  |  |

**Post-critical region (Spillover)**

Model:

*(log)rt ~ group + grammaticality + markedness + group:grammaticality + group:markedness + grammaticality:markedness + group:grammaticality:markedness + (1 | subject) + (1 | item)*

**Table 2.2** Model output.

| **Model output for RTs in the SPRT – Post-critical region spillover** | | | | |  |
| --- | --- | --- | --- | --- | --- |
| *Effect* | *df* | *Chisq* | *p* |  |  |
| group | 1 | 24.64 | **<.001** | ******* |  |
| grammaticality | 1 | 314.39 | **<.001** | ******* |  |
| markedness | 1 | 6.50 | **.011** | * |  |
| group:grammaticality | 1 | 40.73 | **<.001** | ******* |  |
| group:markedness | 1 | 1.18 | .277 |  |  |
| grammaticality:markedness | 1 | 16.83 | **<.001** | ******* |  |
| group:grammaticality:markedness | 1 | 7.16 | **.007** | ****** |  |
| Signif. codes: 0 ‘***’, 0.001 ‘**’, 0.01 ‘*’, 0.05 ‘+’, 0.1 ‘ ’ 1 |  |  |  |  |  |

**Heritage group**

**Critical region (Verb)**

Model:

*(log)rt ~ grammaticality + markedness + proficiency + HL_home + HL_social + bilingualism + grammaticality:markedness + grammaticality:proficiency + grammaticality:HL_home + grammaticality:HL_social + grammaticality:bilingualism + markedness:proficiency + markedness:HL_home + markedness:HL_social + markedness:bilingualism + grammaticality:markedness:proficiency + grammaticality:markedness:HL_home + grammaticality:markedness:HL_social + grammaticality:markedness:bilingualism + (1| subject) + (1| item)*

**Table 2.3** Model output.

| **Model output for RTs in the SPRT – Critical region verb – Heritage** | | | | |
| --- | --- | --- | --- | --- |
| *Effect* | *df* | *Chisq* | *p* |  |
| grammaticality | 1 | 2.19 | .139 |  |
| markedness | 1 | 17.40 | **<.001** | ******* |
| proficiency | 1 | 1.91 | .167 |  |
| HL_home | 1 | 1.67 | .197 |  |
| HL_social | 1 | .91 | .340 |  |
| bilingualism | 1 | 1.28 | .258 |  |
| grammaticality:markedness | 1 | 1.13 | .287 |  |
| grammaticality:proficiency | 1 | 2.40 | .122 |  |
| grammaticality:HL_home | 1 | .13 | .720 |  |
| grammaticality:HL_social | 1 | 2.21 | .137 |  |
| grammaticality:bilingualism | 1 | 2.91 | .088 | + |
| markedness:proficiency | 1 | 4.00 | **.46** | ***** |
| markedness:HL_home | 1 | 17.89 | **<.001** | ******* |
| markedness:HL_social | 1 | .18 | .669 |  |
| markedness:bilingualism | 1 | 1.43 | .231 |  |
| grammaticality:markedness:proficiency | 1 | .02 | .890 |  |
| grammaticality:markedness:HL_home | 1 | 1.07 | .300 |  |
| grammaticality:markedness:HL_social | 1 | .43 | .512 |  |
| grammaticality:markedness:bilingualism | 1 | .07 | .785 |  |
| Signif. codes: 0 ‘***’, 0.001 ‘**’, 0.01 ‘*’, 0.05 ‘+’, 0.1 ‘ ’ |  |  |  |  |

**Post-critical region (Spillover)**

Model:

*(log)rt ~ grammaticality + markedness + proficiency + HL_home + HL_social + bilingualism + grammaticality:markedness + grammaticality:proficiency + grammaticality:HL_home + grammaticality:HL_social + grammaticality:bilingualism + markedness:proficiency + markedness:HL_home + markedness:HL_social + markedness:bilingualism + grammaticality:markedness:proficiency + grammaticality:markedness:HL_home + grammaticality:markedness:HL_social + grammaticality:markedness:bilingualism + (1| subject) + (1| item)*

**Table 2.4** Model output.

| **Model output for RTs in the SPRT – Post-critical region spillover – Heritage** | | | | |
| --- | --- | --- | --- | --- |
| *Effect* | *df* | *Chisq* | *p* |  |
| grammaticality | 1 | 15.13 | **<.001** | ******* |
| markedness | 1 | 3.03 | **.082** | + |
| proficiency | 1 | .10 | .748 |  |
| HL_home | 1 | .97 | .324 |  |
| HL_social | 1 | 1.38 | .240 |  |
| bilingualism | 1 | .50 | .479 |  |
| grammaticality:markedness | 1 | .07 | .792 |  |
| grammaticality:proficiency | 1 | .45 | .502 |  |
| grammaticality:HL_home | 1 | 8.99 | **.003** | ****** |
| grammaticality:HL_social | 1 | .00 | .966 |  |
| grammaticality:bilingualism | 1 | .29 | .587 |  |
| markedness:proficiency | 1 | .01 | .904 |  |
| markedness:HL_home | 1 | .09 | **.761** |  |
| markedness:HL_social | 1 | 6.63 | **.010** | ***** |
| markedness:bilingualism | 1 | 4.57 | **.033** | ***** |
| grammaticality:markedness:proficiency | 1 | 1.07 | .300 |  |
| grammaticality:markedness:HL_home | 1 | 1.32 | .250 |  |
| grammaticality:markedness:HL_social | 1 | .11 | .737 |  |
| grammaticality:markedness:bilingualism | 1 | 2.57 | .109 |  |
| Signif. codes: 0 ‘***’, 0.001 ‘**’, 0.01 ‘*’, 0.05 ‘+’, 0.1 ‘ ’ |  |  |  |  |

**3. Model specifications for the statistical analyses (GJT)**

**Between groups**

Model:

*accuracy ~ group + grammaticality + markedness + group:grammaticality + group:markedness + grammaticality:markedness + group:grammaticality:markedness + (grammaticality+markedness | subject) + (grammaticality | item)*

**Table 3.1** Model output.

| **Model output for accuracy in the GJT between groups** | | | | |  |
| --- | --- | --- | --- | --- | --- |
| *Effect* | *df* | *Chisq* | *p* |  |  |
| group | 1 | 12.98 | **<.001** | ******* |  |
| grammaticality | 1 | 12.20 | **<.001** | ******* |  |
| markedness | 1 | .08 | .780 |  |  |
| group:grammaticality | 1 | .16 | **.691** |  |  |
| group:markedness | 1 | 4.94 | **.026** | ***** |  |
| grammaticality:markedness | 1 | .94 | **.331** |  |  |
| group:grammaticality:markedness | 1 | .05 | .816 |  |  |

**Heritage group**

Model:

*accuracy ~ grammaticality + markedness + proficiency + HL_home + HL_social + bilingualism + grammaticality:markedness + grammaticality:proficiency + grammaticality:HL_home + grammaticality:HL_social + grammaticality:bilingualism + markedness:proficiency + markedness:HL_home + markedness:HL_social + markedness:bilingualism + grammaticality:markedness:proficiency + grammaticality:markedness:HL_home + grammaticality:markedness:HL_social + grammaticality:markedness:bilingualism + (1 | subject) + (1 | item)*

**Table 3.2** Model output.

| **Model output for accuracy in the GJT - Heritage** | | | | |
| --- | --- | --- | --- | --- |
| *Effect* | *df* | *Chisq* | *p* |  |
| grammaticality | 1 | 6.85 | **.009** | ****** |
| markedness | 1 | 2.63 | **.105** |  |
| proficiency | 1 | 15.78 | **<.001** | ******* |
| HL_home | 1 | .58 | .447 |  |
| HL_social | 1 | .12 | .733 |  |
| bilingualism | 1 | .07 | .793 |  |
| grammaticality:markedness | 1 | .03 | .868 |  |
| grammaticality:proficiency | 1 | .34 | .558 |  |
| grammaticality:HL_home | 1 | 3.98 | **.046** | ***** |
| grammaticality:HL_social | 1 | .01 | .942 |  |
| grammaticality:bilingualism | 1 | 2.41 | .120 |  |
| markedness:proficiency | 1 | .13 | .723 |  |
| markedness:HL_home | 1 | 1.16 | **.281** |  |
| markedness:HL_social | 1 | .42 | .517 |  |
| markedness:bilingualism | 1 | .79 | .373 |  |
| grammaticality:markedness:proficiency | 1 | 4.95 | **.026** | ***** |
| grammaticality:markedness:HL_home | 1 | 2.64 | .104 |  |
| grammaticality:markedness:HL_social | 1 | 4.70 | **.030** | ***** |
| grammaticality:markedness:bilingualism | 1 | 4.17 | **.041** | ***** |
